# PDIA4 confers resistance to ferroptosis via induction of ATF4/SLC7A11 in renal cell carcinoma

**DOI:** 10.1038/s41419-023-05719-x

**Published:** 2023-03-11

**Authors:** Lichun Kang, Dekun Wang, Tianyu Shen, Xuan Liu, Bo Dai, Donghui Zhou, Huan Shen, Junbo Gong, Gang Li, Yuanjing Hu, Peng Wang, Xue Mi, Yuying Zhang, Xiaoyue Tan

**Affiliations:** 1 Department of Pathology, School of Medicine, Nankai University, Tianjin, China; 2 Department of Clinical Laboratory, Tianjin First Central Hospital, Tianjin, China; 3 School of Chemical Engineering and technology, Tianjin University, Tianjin, China; 4 Urology Division, The Second Hospital of Tianjin Medical University, Tianjin, China; 5 Department of Gynecologic Oncology, Tianjin Central Hospital of O & G, Tianjin, China; 6 Nankai University Affiliated Eye Hospital, Tianjin, China

**Keywords:** Renal cell carcinoma, Mechanisms of disease

## Abstract

The prognosis of renal cell carcinoma (RCC) remains poor due to metastases and resistance to chemotherapy. Salinomycin (Sal) exhibits the potential of antitumor, while the underlying mechanism is not completely clear. Here, we found that Sal induced ferroptosis in RCCs and identified Protein Disulfide Isomerase Family A Member 4 (PDIA4) as a mediator of Sal’s effect on ferroptosis. Sal suppressed PDIA4 by increasing its autophagic degradation. Downregulation of PDIA4 increased the sensitivity to ferroptosis, while ectopic overexpression of PDIA4 conferred ferroptosis resistance to RCCs. Our data showed that downregulation of PDIA4 suppressed activating transcription factor 4 (ATF4) and its downstream protein SLC7A11 (solute carrier family 7 member 11), thereby aggravating ferroptosis. In vivo, the administration of Sal promoted ferroptosis and suppressed tumor progress in the xenograft mouse model of RCC. Bioinformatical analyses based on clinical tumor samples and database indicated a positive correlation exists between PDIA4 and PERK/ATF4/SLC7A11 signaling pathway, as well as the malignant prognosis of RCCs. Together, our findings reveal that PDIA4 promotes ferroptosis resistance in RCCs. Treatment of Sal sensitizes RCC to ferroptosis via suppressing PDIA4, suggesting the potential therapeutical application in RCCs.

## Introduction

The incidence of renal cell carcinoma (RCC) is continuously increasing, affecting over 400,000 individuals worldwide per year [1]. Although RCC can be successfully treated with surgical or ablative strategies if identified early, up to a third of patients will present with or develop metastases that are associated with a poor prognosis [2]. In addition, resistance to new targeted therapeutics generally develops within months to years. Exploring novel treatment agents and identifying potential targets are urgently needed.

Salinomycin (Sal), a monocarboxylic polyether ionophore antibiotic, did not gain attention in the field of cancer research until 2009. Gupta et al. revealed its antitumor property through high-throughput screening of 16,000 compound libraries. They also identified that the antitumor effect of Sal attributed to targeting cancer stem cells (CSCs) [3]. Afterward, other mechanisms of Sal’s action were successively revealed, including sensitization of multidrug resistance, induction of DNA damage and autophagy, anti-angiogenesis, as well as inhibition of M2 polarization of tumor-associated macrophages [4–6]. Nevertheless, the full-scale framework of Sal in the treatment of cancer has not been established yet.

Ferroptosis is a newly defined form of regulated cell death, characterized by iron-dependent accumulation of lipid hydroperoxides to lethal levels [7]. Triggering ferroptosis emerges as a potential strategy for cancer treatment, especially in case of resistance to chemotherapy. Zou et al. demonstrated an intrinsic vulnerability to ferroptosis of ccRCC (clear cell RCC), which owed to its unique GPX4-dependent metabolic state [8]. Yang and colleagues found that activation of TAZ, the effector of the Hippo signaling pathway, regulates the ferroptosis susceptibility of RCC through increasing accumulation of lipid reactive oxygen species [9]. All these findings highlight the therapeutic approaches aiming at ferroptosis in RCC.

Endoplasmic reticular (ER) stress refers to the imbalance between protein folding load and ER protein folding capacity. Although ER stress can be alleviated when unfolded protein response (UPR) responds properly, it triggers death if the stimuli persist [10]. Regulatory signaling of UPR extensively links with diverse regulated cell death signaling pathways through mechanisms that are still being totally unraveled [11, 12]. Protein disulfide isomerase A member 4 (PDIA4) belongs to the PDA family, a group of ER enzymes responsible for the formation, breakage, and rearrangement of protein disulfide bonds, thus mediating oxidative protein folding [13]. Overexpression of PDIA4 is observed in cisplatin-resistant non-small cell lung cancer [14] and emerges as a novel regulator of cancer growth [15]. The role of PDIA4 in ferroptosis is completely undefined yet.

Herein, we demonstrate the pro-ferroptosis effect of Sal in RCC and identify PDIA4 as the target of its function. What’s more, PDIA4 suppresses ferroptosis through ER stress regulatory signaling pathway PERK/ATF4 and the downstream protein SLC7A11. Our findings reveal a novel mechanism on the tumoricidal effect of Sal and suggest PDIA4 as a potential prognostic and therapeutic target for RCC.

## Results

### Sal induces ferroptosis in renal cell carcinoma cells

**Fig. 1:**
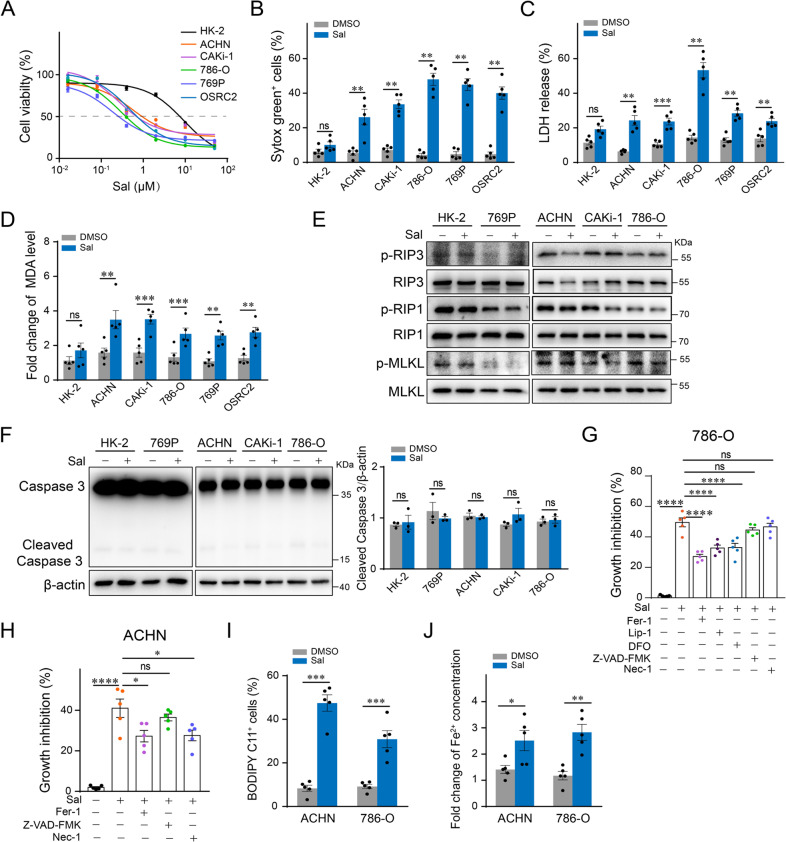
Sal triggers ferroptosis in cultured RCC cell lines. Human proximal tubular cell line HK-2 and RCC cell lines ACHN, CAKi-1, 786-O, 769P, and OSRC2 were treated with different concentrations of Sal as indicated for 24 h. **A** Dose-response viability curves of RCCs and HK-2 cells treated with Sal. **B** Effect of 2 µM Sal against HK-2 and RCC cells measured by Sytox green-staining and **C** LDH release assay. **D** In vitro inductive effect of 2 µM Sal on lipid peroxidation measured by MDA assay. HK-2, 769P, ACHN, CAKi-1, and 786-O cells were treated with 2 µM Sal separately. **E** Expression of RIP3, RIP1, MLKL, and phosphorylated RIP3, RIP1, MLKL analyzed by western blot. **F** Caspase-3 and cleaved caspase-3 analyzed by western blot. The left panel shows the representative images, and the right panel the quantification normalized with b-actin. 786-O cells and ACHN cells were treated with 2 µM Sal for 24 h, respectively. **G**, **H** Cell viability assay in the presence or absence of the indicated inhibitors. **I** Flow cytometry analysis showing lipid ROS by means of fluorogenic reaction with BODIPY 589/591 C11. **J** Assay of ferrous ion. Data are present as mean ± S.E.M. and obtained from at least three independent experiments. *P* values were calculated using Student’s *t*-test in (**I**), (**J**), and one-way ANOVA in (**B**), (**C**), (**D**), (**F**), (**G**), and (**H**). *P < 0.05; **P < 0.01; ***P < 0.001; ****P < 0.0001.

In order to identify the effect of Sal on cell survival, we first examined the IC50 value of Sal against control renal tubular cell HK-2 and various RCC cell lines. The results showed that IC50 of Sal displayed ~10-fold higher potency against RCC cells than that of HK-2 (Fig. 1A). Dye of Sytox Green and LDH release assay showed that 2 μM of Sal exhibited significant cytotoxicity and resulted in substantial cell death in RCCs (Fig. 1B, C). It has been reported that RCCs are intrinsically susceptible to ferroptosis. We next evaluated whether Sal induces ferroptosis in RCCs. End-product of lipid peroxidation MDA was used to evaluate the level of lipid peroxidation. Our data showed that Sal increased the level of MDA in RCC cells without significant influence in HK-2 cells (Fig. 1D). Western blot assay showed that Sal did not result in significant changes in the level of phosphorylated RIP3, RIP1, and MLKL, as well as cleaved caspase-3 (Fig. 1E, F). Inhibitors for ferroptosis, apoptosis, and necroptosis were separately applied to identify the style of Sal-induced cell death in RCC cells. Our results showed that ferroptosis inhibitors Ferrostatin-1, Liproxstatin-1, and Deferoxamine exhibited greater protective effect against Sal-induced cell death than the pan-apoptosis inhibitor or necroptosis inhibitor, suggesting the involvement of ferroptosis (Fig. 1G, H). Results of the C11 BODIPY assay confirmed that Sal stimulated the upregulation of lipid peroxidation (Fig. 1I). In addition, the detection of ferrous ions showed that Sal increased the level of intracellular iron (Fig. 1J), supporting that Sal induces ferroptosis in RCC cells.

We also tested the effect of Sal on ferroptosis in breast cancer cell lines MCF-7, MDA-MB-231, SK-BR3, and control breast epithelial cells MCF-10A. Although less significant, it can be observed that breast cancer cells were more sensitive to Sal-induced death compared with control (Supplementary Fig. S1A). Similarly, Sal increased the level of MDA in MDA-MB-231 cells (Supplementary Fig. S1B). Inhibitors of ferroptosis exhibited a greater extent of protective effect on the Sal-induced cell death than inhibitors of necroptosis or apoptosis (Supplementary Fig. S1C). Altogether, our data demonstrate that Sal induces ferroptosis in cancer cells.

### Sal suppresses PDIA4 by promoting its autophagic degradation

**Fig. 2:**
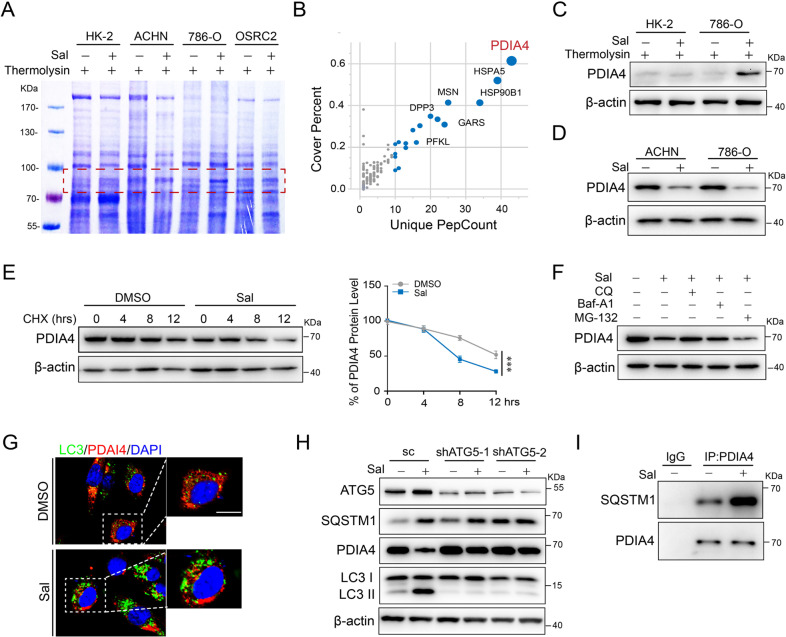
Sal induces autophagic degradation of PDIA4. HK-2 and RCC cell lines ACHN, 786-O, and OSRC2 were treated with DMSO or Sal (2 µM), and lysates were subjected to thermolysin digestion. **A** Coomassie blue staining. **B** Enrichment of proteins in the protected band (red frame in (**A**)) revealed by mass spectrometry analysis. **C** Western blot assay of cell lysates using antibodies against PDIA4. 786-O and ACHN cells were treated with DMSO or Sal (2 µM). **D** Western blot assay of cell lysates using antibodies against PDIA4. The volume of β-actin was used as the loading control. 786-O cells were treated with DMSO or Sal (2 µM) and subjected to protein synthesis inhibitor CHX as indicated. **E** Western blotting assay of cell lysates using antibodies against PDIA4 (left). The curve of protein degradation rate (right). Data are present as mean ± S.E.M., *n* = 5. *P* values were calculated using Student’s *t*-test. ***P < 0.001. 786-O cells were treated with DMSO or Sal (2 µM) in the presence of proteasome inhibitor MG-132 or autophagy inhibitor Baf-A1 or CQ. **F** Western blot assay of cell lysates using antibodies against PDIA4. 786-O cells were treated with DMSO or Sal (2 µM). **G** Immunofluorescence staining using antibodies against LC3 (green) and PDIA4 (red). Scale bar: 10 μm. Stable ATG5-silencing down or scramble control 786-O cells were treated with DMSO or Sal (2 µM). **H** Western blot assay of cell lysates using antibodies against ATG5, SQSTM1, PDIA4, or LC3. **I** Co-immunoprecipitation assay of cell lysates in Sal-treated 786-O cells. IP was performed using antibodies against PDIA4 and immunoblotting with antibodies against SQSTM1.

We then explored the mechanism of the inductive role of Sal in ferroptosis in RCC cells. Drug affinity responsive target stability (DARTS) assay was used to identify the molecular target of Sal, which takes advantage of a reduction in the thermolysin susceptibility of the target protein upon drug binding. We found a strong protected band at ~85 kDa in the proteolyzed extracts of Sal-treated 786-O cells, whereas no detectable difference was observed in the extracts of Sal-treated HK-2 cells (Fig. 2A). Examination of the protected band and the matching gel region of the control lane by mass spectrometry identified PDIA4 as the binding protein at high abundance in the Sal-treated RCC sample (Fig. 2B). Western blot assay demonstrated the binding of Sal and PDIA4 in the presence of thermolysin (Fig. 2C), and confirmed reduction of PDIA4 upon Sal treatment (Fig. 2D).

Considering no significant change of PDIA expression at the mRNA level after treatment of Sal (data not shown), we then detected the degradation of PDIA4 upon the treatment of Sal. We found that Sal accelerated the degradation rate of PDIA4 in RCCs (Fig. 2E). Autophagy inhibitor Chloroquine and Bafilomycin A1, not proteasome inhibitor MG-132, could rescue the inhibition of PDIA4 induced by Sal, suggesting the role of autophagic degradation (Fig. 2F). Immunofluorescence staining revealed that Sal induced increase of LC3 and decrease of PDIA4 (Fig. 2G). Consistently, the western blot assay showed that Sal increased the level of LC3II and suppressed PDIA4. Suppression of autophagy via silencing down ATG5 rescued the Sal-induced inhibition of PDIA4. Interestingly, Sal induced SQSTM1 in 786-O cells (Fig. 2H). Immunoprecipitation analysis confirmed Sal promoted the interaction of SQSTM1 and PDIA4 (Fig. 2I). Collectively, these findings indicate that autophagic degradation involves in the Sal-induced decrease of PDIA4.

### Downregulation of PDIA4 mediates the effect of Sal on ferroptosis in RCC

**Fig. 3:**
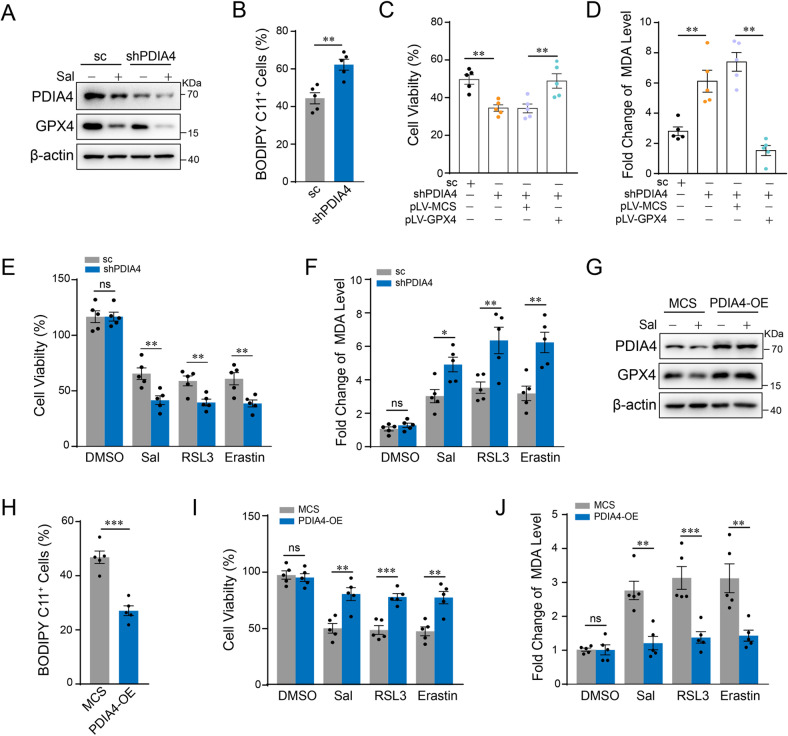
PDIA4 regulates the sensitivity of RCCs to ferroptosis. Stable PDIA4-silencing-down or scramble control 786-O cells were treated with Sal (2 µM). **A** Western blot assay of cell lysates using antibodies against PDIA4 and GPX4. **B** Flow cytometry analysis showing lipid ROS by means of fluorogenic reaction with BODIPY 589/591 C11 in stable PDIA4-silencing-down or scramble control 786-O cells. Stable PDIA4-silencing-down or scramble control 786-O cells were treated with Sal (2 µM) and transfected with GPX4-overexpressing or control plasmid. **C** Cell viability assay and **D** level of lipid peroxidation measured by MDA assay. Stable PDIA4- or scramble control-silencing down 786-O cells were treated with Sal (2 µM) or separate ferroptosis inducers, RSL3 (1 µM) and erastin (2 µM) for 24 h. **E** Cell viability assay and **F** level of lipid peroxidation measured by MDA assay. Stable PDIA4- or control MCS-overexpressing 786-O cells were treated with Sal (2 µM) or separate ferroptosis inducers, RSL3 (1 µM) and erastin (2 µM) for 48 h. **G** Western blot assay of cell lysates using antibodies against PDIA4 and GPX4. **H** Flow cytometry analysis showing lipid ROS by means of fluorogenic reaction with BODIPY C11. **I** Cell viability assay and **J** level of lipid peroxidation measured by MDA assay. Data are present as mean ± S.E.M., *n* = 5 independent repeats. Student’s *t*-test was performed in (**B**) and (**H**). One-way ANOVA analysis was performed in (**C**), (**D**), (**E**), (**F**), (**I**), and (**J**). *P < 0.05; **P < 0.01; ***P < 0.001.

PDIA4 belongs to the PDIs family and acts as a disulfide isomerase or protein chaperone in ER stress. We first compared the expression of PDIA4 in different RCC cell lines and control HK-2 cells, as well as in the RCC tumor and adjacent normal tissues. Data showed that expression of PDIA4 was increased in RCCs compared with the control, both in the RCC cell lines and human tumor tissues (Supplementary Fig. S2). Furthermore, we constructed stable PDIA4-silencing down and PDIA4-overexpressing 786-O cell lines. Two-dimensional colony formation assay showed that silencing down of PDIA4 suppressed while overexpression of PDIA4 promoted the growth of 786-O cells (Supplementary Fig. S3). We then detected the role of PDIA4 in Sal-induced ferroptosis. Western blot showed that Sal inhibited GPX4, a central regulator of ferroptosis, while silencing down of PDIA4 aggravated this effect (Fig. 3A). Results of C11 Bodipy assay showed the increased lipid peroxidation in PDIA4-silencing down 786-O cells compared with control (Fig. 3B). Cell viability and MDA assay showed that overexpression of GPX4 rescued the aggravated Sal-induced cell death and increase of lipid peroxidation in the PDIA4-silencing down cells (Fig. 3C, D).

Next, we examined the effect of PDIA4 downregulation on ferroptosis induced by erastin and RSL3. Data from CCK-8 and MDA assay demonstrated that the downregulation of PDIA4 aggravated the cell death induced by erastin and RSL3 (Fig. 3E, F). Consistent results were obtained from the experiments on PDIA4 overexpression. Sal-induced decrease of GPX4 was protected by overexpression of PDIA4 (Fig. 3G). Results of the C11 Bodipy assay showed less Sal-induced lipid peroxidation in PDIA4-overexpressing 786-O cells compared with the control (Fig. 3H). CCK-8 cell viability and MDA assay showed that overexpression of PDIA4 promotes the resistance to ferroptosis induced either by Sal or by erastin or RSL3 (Fig. 3I, J). Therefore, our data suggest that PDIA4 promotes ferroptosis resistance and involves in the pro-ferroptotic effect of Sal in RCCs.

### PDIA4 targets SLC7A11 through PERK/ATF4 pathway

**Fig. 4:**
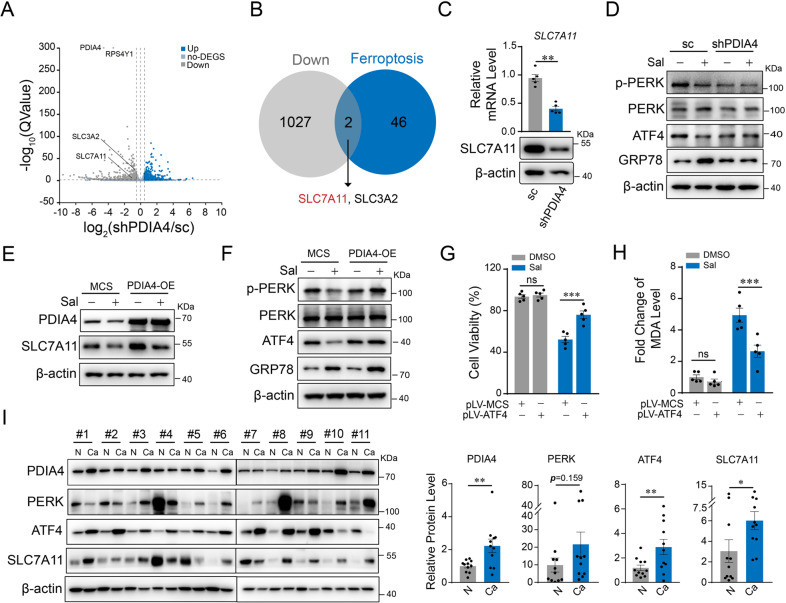
Downregulation of PDIA4 suppresses ATF4/SLC7A11/GPX4 in RCCs. RNA-seq was performed in stable PDIA4-silencing-down 786-O cells, with scramble control 786-O cells as control. **A** Scatter plot of differential expression genes. **B** Overlay of downregulated genes (shPDIA4 vs scramble control) with known regulatory genes in ferroptosis. **C** Real-time PCR and western blot assay for *SLC7A11* expression in stable PDIA4-silencing-down or scramble control 786-O cells. Stable PDIA4-silencing-down or scramble control 786-O cells were treated with Sal (2 µM). **D** Western blot assay of cell lysates using antibodies against p-PERK/PERK and ATF4. Stable PDIA4- or control MCS-overexpressing 786-O cells were treated with Sal (2 µM) for 24 h. **E**, **F** Western blot assay of cell lysates using antibodies against SLC7A11, PDIA4, phosphorylated PERK, and ATF4. 786-O cells were transfected with ATF4 overexpressing plasmid or control plasmid in the presence with or without Sal (2 µM). **G** Cell viability assay and **H** level of lipid peroxidation measured by MDA assay. **I** Western blot assay of PDIA4, PERK, ATF4, and SLC7A11 in cancerous and adjacent normal tissue resected from patients of RCC, and the statistical analyses. Data are present as mean ± S.E.M., *n* = 5. Student’s *t*-test was performed in (**C**) and (**I**), and one-way ANOVA analysis was performed in (**G**) and (**H**). *P < 0.05; **P < 0.01; ***P < 0.001.

RNA sequencing and differential expression genes assay were used to explore the downstream signal of PDIA4 (Fig. 4A). Through the intersection of downregulated genes upon PDIA4-silencing down and ones known as regulatory genes of ferroptosis, we screened out SLC7A11 (Fig. 4B). Real-time PCR and western blot assay confirmed the inhibition of SLC7A11 when silencing down PDIA4, at both the mRNA and protein level (Fig. 4C). PERK/ATF4 is one of the central signaling pathways involved in the ER stress response. Moreover, previous studies showed that PDI family member effected on ER stress via different transducers of UPR [16]. We then examined the active state of PERK/ATF4 signaling, as well as a critical chaperone in ER stress GRP78. Western blot showed that either Sal or silencing down of PDIA4 inhibited phosphorylation of PERK and its major effector ATF4, while increased expression of GRP78 (Fig. 4D). Overexpression of PDIA4 rescues the Sal-induced suppression of SLC7A11 (Fig. 4E), as well as Sal-induced decrease of phosphorylated PERK and ATF4 (Fig. 4F). Similar results were observed in Sal-treated RCC cell line ACHN (Supplementary Fig. S4). In addition, overexpression of ATF4 alleviated Sal-induced cell death detected by CCK-8 and MDA assay (Fig. 4G, H).

We then collected resected tumors and corresponding paracancerous samples from patients with RCCs and performed the expressional analysis on PDIA4, PERK, ATF4, and SLC7A11. We found that PDIA4, PERK, ATF4, and SLC7A11 exhibited a tendency to increase in the tumors compared with control (Fig. 4I). Expression of the other two UPR signaling pathways in ER stress, IRE-1α and ATF6 exhibited no difference between the RCC tumor tissue and normal control (Supplementary Fig. S5A). Similar results were obtained in cultured HK-2 cells and RCC cells, that is, the expression of ATF4 was increased while there was no change in the expression of IRE-1α and ATF6 compared with the control (Supplementary Fig. S5B, C). Collectively, our findings indicate that inhibition of PDIA4 suppresses PERK/ATF4 signaling pathway and consequent ferroptosis regulator SLC7A11.

### Sal inhibits tumor growth and promotes ferroptosis in vivo

**Fig. 5:**
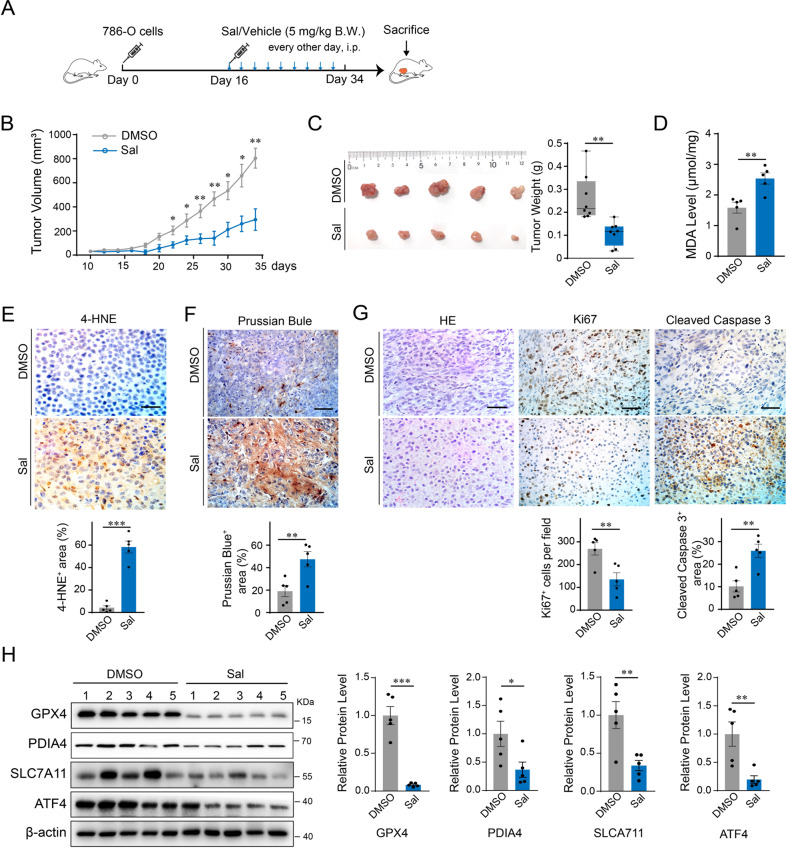
Sal suppresses the growth of RCC by promoting ferroptosis in vivo. 2 × 10^6^ 786-O cells were administrated subcutaneously to BALB/c nude mice to establish xenograft mouse models. Animals were randomly divided into Sal treatment and vehicle control groups (*n* = 8), with the administration of Sal (5 mg/kg BW, intraperitoneally, every other day) or solvent vehicle from the day when the size of the tumor reaches 50 mm^3^. **A** Schematic diagram of in vivo experimental design. **B** Tumor growth curve. **C** Representative images of isolated tumor (left) and the result of statistical analysis on the size data (right). **D** Level of lipid peroxidation measured by MDA assay in the tumor homogenates. **E** Representative images (up) and statistic data (bottom) of 4-HNE assay and **F** Representative images (up) and statistic data (bottom) of Prussian blue staining. **G** Representative images of H&E, IHC staining of Ki67, and cleaved caspase-3 staining (up) and statistic data (bottom). Scale bar: 50 µm. **H** Western blot analysis on the tumor tissue homogenates by using antibodies against PDIA4, ATF4, SLC7A11, and GPX4. The right panel is the result of the statistical analysis of the integral optical density of separate bands. Data are present as mean ± S.E.M., *n* = 5. *P* values were calculated using a two-tailed unpaired Student’s *t*-test. *P < 0.05; **P < 0.01; ***P < 0.001.

To validate the pro-ferroptotic effects of Sal in vivo, we established subcutaneous xenograft mouse models of RCC using 786-O cells in nude BALB/c mice (Fig. 5A). Tumor growth was significantly suppressed in the group of Sal compared with vehicle control (Fig. 5B, C). Accumulation of lipid peroxidation in the Sal group compared with control as shown by MDA level (Fig. 5D). 4-HNE staining and Perls’ Prussian blue staining demonstrated increased intracellular ROS and iron in the Sal group, indicating increased ferroptosis (Fig. 5E, F). IHC staining of Ki67 and cleaved caspase-3 showed a decrease in proliferation and an increase in cell death in the Sal-treated group (Fig. 5G). In cultured 786-O cells, administration of Sal decreases GPX4, as well as PDIA4, ATF4, and SLC7A11 (Fig. 5H). Together, these data support that Sal promotes ferroptosis and thus suppresses tumor growth in RCCs.

**Fig. 6:**
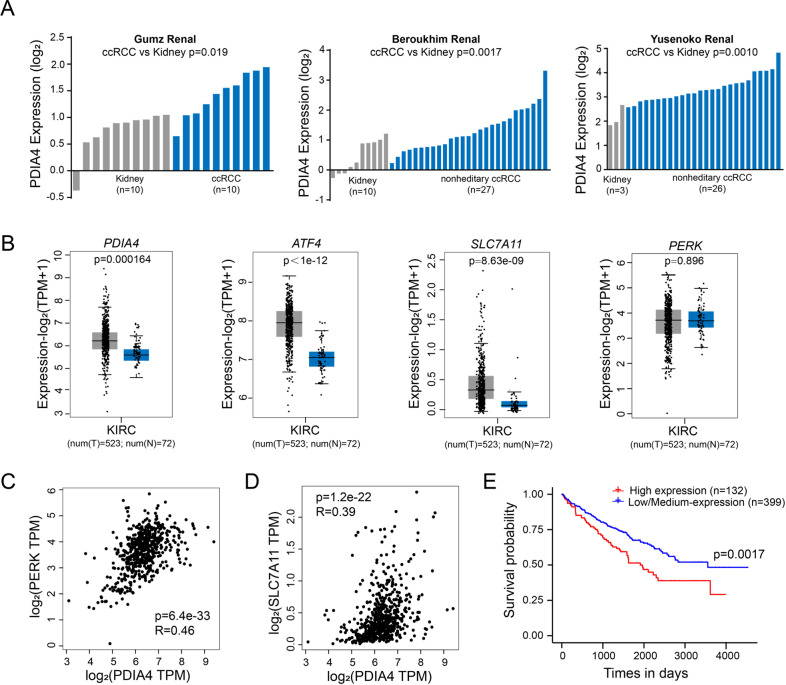
PDIA4 functions as a prognostic index for human renal clear cell carcinoma. **A** Comparison of PDIA4 gene expression between normal kidney tissue and a cancerous sample of ccRCCs. Data were obtained from Gumz, Beroukhim, and Yusenko database sets, respectively. **B** Comparison of the gene expression data of PDIA4, ATF4, SLC7A11, and PERK between normal and carcinoma tissues obtained from the TCGA database. **C**, **D** Correlation assay on the gene expression data of PDIA4 and PERK or PDIA4 and SLC7A11 in RCC patients obtained from TCGA database. **E** Kaplan–Meier survival plot of PDIA4 high expression and low/medium expression groups. Data were obtained from the TCGA database.

To identify the role of PDIA4 as a prognostic index for RCC, we also performed data-mining analyses from three microarray datasets in the Oncomine database, all of which were analyzed using the Human genome U133A Array. The mRNA level of PDIA4 was enhanced significantly in human RCC samples as compared to normal renal tissues (Fig. 6A). Based on the TCGA database, we compared the mRNA expression of PDIA4, ATF4, SLC7A11, and PERK between RCC tumor and adjacent normal tissue. The result of the analysis revealed the expressional level of PDIA4, ATF4, and SLC7A11 in the RCC tumor is higher than the one in the adjacent normal tissue, respectively (Fig. 6B). Moreover, a positive correlation exists in the expression of PDIA4 with either PERK or SLC7A11 (Fig. 6C, D). Kaplan–Meier survival analysis revealed that a significant difference exists between PDIA4 low/medium and high expression RCC patients (Fig. 6E). Overall, the bioinformation data support the potential role of PDIA4 as a diagnostic index for RCCs, as well as the correlation of PDIA4 with PERK/ATF4/SLC7A11 axis.

**Fig. 7:**
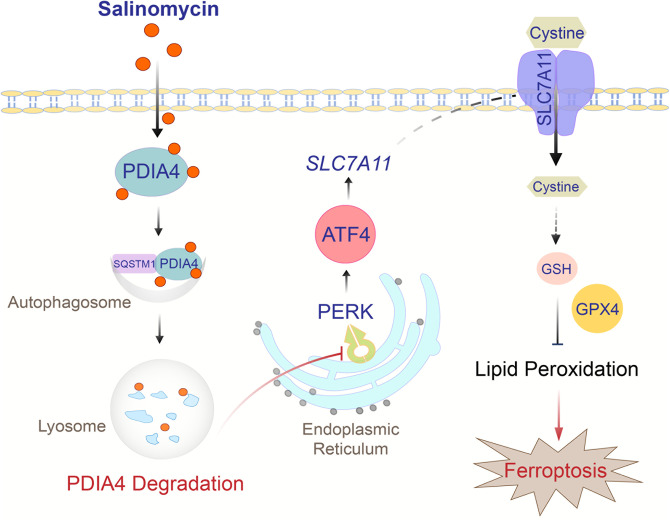
Schematic illustration of the hypothesized mechanism of Sal’s effect on ferroptosis. Sal exhibits pro-ferroptic effect in RCC via downregulating PDIA4. Administration of Sal stimulates autophagic degradation and thus inhibits PDIA4. Downregulation of PDIA4 suppresses ATF4 and SLC7A11, thereby inactivates GPX4 and promotes ferroptosis.

In summary, our data here reveal that Sal promotes autophagic degradation of ER stress-related chaperone PDIA4 and thus induces ferroptosis in RCC. Sal-induced downregulation of PDIA4 confers ferroptosis sensitivity through PERK/ATF4 signaling pathway and its downstream target SLC7A11 (Fig. 7).

## Discussion

Although substantial success has been achieved on selective targeted drug and tumor immunotherapy, the prognosis of RCC remains poor, and thus necessity arises for novel therapeutics [17]. Previous studies identified the anticancer effect of Sal through specific targeting of CSCs and sensitization of therapy-resistant cells [18, 19]. Here, we demonstrate the potential of Sal as a remedy for RCC, as well as reveal a novel mechanism of its anticancer effect of improving ferroptosis sensitivity. Sal suppresses PDIA4, an ER stress-related chaperone, through induction of its autophagic degradation. Downregulation of PDIA4 suppresses PERK/ATF4 signaling and downstream target cystine transporter SLC7A11, which in turn, interrupts the GPX4-mediated detoxification of lipid peroxides and thus facilitates ferroptosis.

Membrane ionophore Sal was regarded solely as an antibiotic until Gupta et al. unveiled its specific toxicity for epithelial CSCs in 2009 [3]. From then on, various studies demonstrated the anticancer potential of Sal in different types of cancer. Although the mechanism remains elusive, the antitumor effect of Sal arouses great interest in the field of oncology. Apart from the removal of CSCs and inhibition of chemotherapy resistance, other attractive characteristics such as anti-angiogenetic activity and effect on EMT have been identified in Sal, implying the diversity and heterogeneity of Sal’s role. Our data here showed that the effectiveness of Sal against RCC cells is about 10 times that of normal renal tubular cells. Consistently, the administration of Sal significantly suppressed tumor growth in the subcutaneous xenograft tumor model of RCC. It is noteworthy that the anticancer potency of Sal in breast cancer cells is not comparable with that in RCC, suggesting the specificity of RCC for the antitumor effect of Sal.

Ferroptosis is a form of regulated cell death characterized by lethal lipid peroxidation [20]. Emerging evidence suggests that ferroptosis participates in the pathogenesis of different diseases, including neurodegenerative diseases, ischemic injury, and neoplastic diseases [21–23]. Thereinto, the tumor-suppressor function of ferroptosis stands out as being potential therapeutics for malignancy [24]. Studies showed that the initiation and execution of ferroptosis are tightly linked to the metabolism of amino acid, iron, and polyunsaturated fatty acid [25]. As it happens, metabolic reprogramming is the prominent feature of RCC, such that this cancer is labeled a metabolic disease [26]. Therefore, it is legitimate to assume that RCC possesses superior sensitivity to ferroptosis. Investigation of metabolic dependencies verified that RCC cells are hypersensitive to the depletion of glutamine or cystine, two amino acids required for glutathione (GSH) synthesis, which in turn leads to GPX4 inhibition and thus ferroptosis [27]. A salient feature of RCC is significant dysregulation of the hypoxia-inducible factor pathway, usually related to the loss of von Hippel-Lindau tumor-suppressor function. Recent studies revealed that HIF-2α activates the expression of lipid droplet-associated protein, thereby enriching polyunsaturated lipids and facilitating ferroptosis [8]. Here, we screened out PDIA4 as a target of Sal in RCC by analyzing the stability of affinity with Sal and identified its role in regulating the sensitivity to ferroptosis in RCCs.

PDIA4 is the largest protein member in the PDI family, which mainly functions as a molecular chaperone and binds to unfolded protein substances [8]. Through catalyzing the formation of disulfide bonds between cysteine residues, PDIA4 is responsible for protein folding and increases along with intensified ER stress response [13]. Increased expression of PDIA4 was observed in various types of cancer, while the detailed mechanisms are in deficiency [28, 29]. Kou et al. reported that PDIA4 inhibits caspases 3 and 7 and therefore facilitates tumor growth [15]. Another study suggests that inhibition of PDIA4 ameliorates cisplatin resistance in lung cancer [14]. Our data here demonstrated the increase of PDIA4 in RCC. Moreover, the level of PDIA4 correlates positively with the malignancy of RCC. Administration of Sal suppresses PDIA4 via binding PDIA4, thereby promoting its lysosomal degradation. Inhibition of PDIA4, pharmaceutically or genetically, sensitizes RCC to ferroptosis.

In this study, we found that the Sal-induced inhibition of PDIA4 does not belong to the transcriptional level but the protein level. Inhibitors of lysosomal degradation could restore the PDIA4 inhibition resulting from Sal. Moreover, Sal increases LC3II as well as the interaction of SQSTM1/p62 with PDIA4, implying that selective autophagy contributes to the decrease of PDIA4. Unexpectedly, SQSTM1/p62 is increased upon the treatment of Sal. Previous studies showed that inhibition of Nrf2 led to the downregulation of the p62 [30, 31]. Sal-induced induction of Nrf2 was observed to begin as early as 3 h after Sal treatment (data not shown), suggesting that Nrf2 regulates the increase of p62. Komatsu et. al reported that p62 competitively interacts with Keap1 and thus gives rise to an accumulation of free Nrf2 and its nuclear translocation [32]. Therefore, it becomes highlighted that p62 is not only a factor that targets specific cargoes for selective autophagy but also a signal contributing to the status of oxidative stress. It is obviously important to clarify the mutual interaction between p62 and Nrf2 in distinct diseases context in the future.

ER stress occurs upon the accumulation of unfolded proteins in the lumen of the ER or distinct insults that disturb the ER’s normal function, which initiates UPR in order to restore ER homeostasis. Paradoxically, the UPR can also induce cell death under severe and/or permanent ER stress. The UPR depends on three signaling pathways, the inositol-requiring enzyme-1α (IRE-1α), the pancreatic ER kinase-like ER kinase (PERK)/ATF4, and the activating transcription factor 6 (ATF6) [33, 34]. The type of mediator as well as the consequence of UPR is highly heterogeneous, varying with the context of pathologic state and origin of cell resource. Mechanisms linking ER stress signaling with cell death are still unraveled. In this study, we found PDIA4 activates PERK/ATF4 pathway in RCC. ATF4 transcriptionally activates various stress-dependent gene expressions, including SLC7A11 [34, 35]. SLC7A11 acts as the catalytic subunit of cystine/glutamate antiporter system X_C_^-^, controlling the transportation of extracellular cystine and thus the production of glutathione (GSH) synthesis [36, 37]. GSH protects against lipid peroxidation and ferroptosis by acting as a rate-limiting substrate for GPX4.

We also detected the level of GRP78 (glucose-regulated protein 78), an essential molecule for the regulation of UPR upon the treatment of Sal. Our data showed that Sal and downregulation of PDIA4 result in the increase of l GRP78. Studies revealed that a load of unfolded protein induces the dislocation of GRP78 from UPR transducers and thus activates distinct UPR signaling pathways. Activation of UPR, in turn, leads to the increase of GRP78, aiming to relieve ER stress [38]. Interestingly, studies suggest PDIA is involved in the regulation of the UPR pathway via distinct mechanisms, including competing with GRP78 for binding IRE-1α or PERK [16]. Here, our results show that the Sal-induced decrease of PDIA4 inhibits PERK-ATF4 signaling, along with the induction of GRP78. Whether PDIA4 effects PERK/ATF4 signaling via competing with GRP78 for binding PERK as well as the mechanism underlying the Sal-induced increase of GRP78 apparently deserves further pursuit.

Herein, we decipher that Sal exhibits anticancer effects in RCC via conferring the ferroptosis sensitivity. Mechanistically, Sal stimulates autophagic degradation and thus inhibits PDIA4. Downregulation of PDIA4 suppresses ATF4 and its target molecule SLC7A11 thereby inactivating GPX4 and promoting ferroptosis. In vivo, a higher level of PDIA4 correlates with a worse prognosis of RCC. In view of the role of ferroptosis in extensive pathophysiological processes of diseases, these insights shed light on novel therapies for RCCs and other ferroptosis-involved diseases.

## Materials and methods

### Cell culture and reagents

Human proximal tubule cell lines HK-2, human kidney cancer cell lines 786-O, 769P, ACHN, and CAKi-1 cells were obtained from American Type Culture Collection (ATCC). Human kidney cancer cell lines OSRC2 was purchased from the National Infrastructure of Cell Line Resource (Beijing, China). HK-2 cells were maintained in Dulbecco’s Modified Eagle Medium/F12 medium (DMEM/F12, 50:50, Biological Industries), supplemented with 10% (v/v) bovine calf serum (Biological Industries) and 1% (v/v) penicillin and streptomycin (Life Technologies). The 786-O, 769P, and OSRC2 cell lines were cultured in the RPMI-1640 medium. The ACHN cell line was cultured in Eagle’s Minimum Essential Medium (Life Technologies), and the CAKi-1 cell line was cultured in McCoy’s 5a Medium Modified (Life Technologies). All cell lines were maintained at 37 °C with 5% CO_2_.

For cell viability assays, cells were treated with Salinomycin (1 μM, HY-15597, MedChem Express, MCE); ferroptosis inducers, Erastin (5 μM, HY-15763, MCE) and RSL3 (0.5 μM, HY-100218A, MCE); cell death inhibitors, Ferrostatin-1 (10 μM, HY-100579, MCE), Liproxstatin-1 (100 nM, HY-12726, MCE), Deferoxamine (100 μM, HY-15760, MCE), Necrostatin-1 (20 μM, HY-15760, MCE), or Z-VAD-FMK (50 μM, HY-166588, MCE). For protein degradation assays, cells were treated with Cycloheximide (20 μg/ml, HY-12320, MCE) for 4 h, 8 h, and 12 h. For autophagy and proteasome-dependent degradation inhibition assays, cells were pretreated with Chloroquine (50 μM, HY-17589A, MCE), Bafilomycin A1 (100 nM, S1413, Selleck), and MG-132 (5 μM, HY-13259, MCE) separately for 2 h, then washed out and treated with Sal for another 24 h.

### Construction of overexpression/knockdown stable cell lines

Full-length complementary DNAs (cDNAs) of human *PDIA4* and *GPX4* were amplified from DNA templates using a standard PCR-based cloning strategy. PCR products were then ligated into a pLV-EF1α-MCS-IRES-Bsd vector (Biosettia) separately. shRNAs against *PDIA4* or *ATG5* were constructed using pLKO.1-TRC cloning vector (Addgene) according to the manufacturer’s protocol. The sequences of the primers used in shRNAs construction are listed in Supplementary Table 1.

Stable cell lines were generated as described previously. Briefly, plasmids used for overexpression or knockdown of the target genes were co-transfected with lentivirus packaging vector mix (Biosettia) into HEK293T cells. After 48 h, cultured medium from transfected cells was collected and filtered with a 0.45-μm filter. Cells were incubated with the filtered medium in the presence of 8 μg/ml polybrene (Sigma-Aldrich) overnight and selected with appropriate antibiotics after 48 h.

### Cell viability measurement and cell death quantification

Relative cell viabilities were determined using Cell Counting Kit-8 (CK04, Dojindo Laboratories, Kumamoto, Japan). Briefly, cells were seeded onto 96-well plates at a density of 5 × 10^3^ per well. The next day, cells were treated as indicated and then exposed to 10 μl CCK-8 reagent (100 μl medium per well) for 2 h at 37 °C, 5% CO2 in an incubator. The absorbance at a wavelength of 450 nm was determined using Clariostar microplate reader (BMG Labtech).

Cell death was measured by Sytox^TM^ green (S34860, Life technology) staining. Cells were treated with appropriate drugs, then trypsinized and collected into a 1.5 ml tube. After washing once with PBS, cells were stained with 1 μM Sytox green for 15 min. Dead (Sytox green^+^) cells were detected by a BD Caliber flow cytometer (BD Biosciences).

### LDH release assay

LDH assays were performed using the LDH Release Assay Kit (C0016, Beyotime) following the manufacturer’s instructions. Briefly, cells were seeded onto 96-well plates and then treated with Sal the next day. After 24 h, plates were centrifuged at 400 g for 5 min. Then, 120 μl supernatant was transferred from each well to a well containing 60 μl reaction buffer, and plates were incubated at room temperature for 30 min. The absorbance was determined on a microplate reader at 490 and 600 nm wavelengths. The 490 nm absorbance was subtracted from the 600 nm absorbance, and the background from the cell culture medium was subtracted from all values.

### BODIPY C11 assay

Cells were seeded in 6-well plates and incubated overnight. The next day, cultured media was replaced with media containing Sal for 24 h. After incubation, cells were harvested by trypsinization, washed, and resuspended in 500 μl PBS containing 5 μM BODIPY^TM^ 589/591 C11 dye (D3861, Invitrogen). Cells were incubated for 30 min at 37 °C. BODIPY C11^+^ cells were determined by the flow cytometer analysis. A minimum of 10,000 cells were counted and analyzed.

### MDA assay

Relative MDA levels in cells and tumor tissues were determined by Lipid Peroxidation MDA Assay Kit (S0131M, Beyotime). Briefly, cells or tissues were homogenized with RIPA lysis buffer. Protein samples were adjusted to the same concentration with lysis buffer and then subjected to react with a thiobarbituric acid solution. After incubation for 15 min at 100 °C, the absorbance at 532 nm was read on a microplate reader.

### Binding protein identification

Binding protein identification was performed by a method, drug affinity responsive target stability (DARTS), as previously described [39]. Briefly, 786-O cells were treated with Salinomycin or DMSO for 40 min, then lysed with RIPA lysis buffer. After centrifugation for 10 min, lysates were adjusted to the same protein concentration and final volume with reaction buffer (50 mM Tris-HCl (pH 8.0), 50 mM NaCl, 10 mM CaCl_2_). All steps were performed on ice to prevent premature protein degradation. Then, 50 μg of lysate was proteolyzed using 100 ng thermolysine for 10 min. To terminate the proteolysis reaction, 0.5 M EDTA (pH 8.0) was applied to each sample at a ratio of 1:10. All samples were then boiled in 5×SDS-PAGE loading buffer and separated by 10% Tris-HCl SDS-PAGE followed by Coomassie blue staining. Protein bands enriched by Salinomycin treatment were then subjected to in-gel trypsin digestion followed by mass spectrometry analysis.

### Western blot and immunoprecipitation assays

Whole-cell lysates from cells and tissues were isolated using RIPA cell lysis buffer with protease inhibitors (MCE). Protein concentration was assayed using Pierce^TM^ BCA protein assay (23225, Thermo Fisher Scientific). Primary antibodies were incubated overnight at 4 °C, and specific proteins were visualized by Pierce^TM^ ECL plus (32109, Thermo Fisher Scientific). Band intensities were measured using Image (Tannon, Shanghai) and normalized to β-actin. Primary antibodies used in this study are as follows: PDIA4 (14712-1-AP, Proteintech), Caspase-3 (9664S, Cell Signaling Technology), Necroptosis antibody sampler kit (98110T, Cell Signaling Technology), LC3 (M152-3, MBL Life Science), SQSTM1 (ab56416, Abcam), ATG5 (ab108327, Abcam), GRP78 (66574-1-Ig, Proteintech), PERK (24390-1-AP, Proteintech), p-PERK (29546-1-AP, Proteintech), GPX4 (ab125066, Abcam), ATF4 (10835-1-AP, Proteintech), SLC7A11 (26864-1-AP, Proteintech), and β-actin (sc-47778, Santa Cruz).

For the immunoprecipitation assay, 786-O cells that overexpress PDIA4 were collected in RIPA lysis buffer. Supernatants were immune-precipitated with 2 μg PDIA4 antibody overnight and then incubated with protein G agarose beads for another 5 h at 4 °C. SQSTM1 were then detected as described for western blot analyses.

### Quantitative real-time PCR analysis

Total RNA was extracted from cells by using TRIzol reagent (Invitrogen) according to the manufacturer’s protocol. RNA (1 μg) was transcribed to cDNA with a Reverse Transcriptase transcription kit (Transgene). Quantitative real-time PCR was performed with a qPCR kit for SYBR Green I (Transgene) in a LightCycler 96 system (Roche). *β-actin* was used as the housekeeping gene. The relative gene expression was calculated following the 2^−△△Ct^ method. The sequences of the primers used in RT-PCR experiments are listed in Supplementary Table 2.

### H&E, immunohistochemistry, and immunofluorescence staining

Formalin-fixed and paraffin-embedded specimens were collected and sectioned at 5 μm. For H&E staining, tissue sections were stained with hematoxylin and eosin (ZSGB-BIO, Beijing, China). For immunohistochemistry staining, after being treated with antigen retrieval solution (Tris-EDTA buffer, pH 9.0), tissue sections were incubated with PDIA4 primary antibody, biotin-conjugated secondary antibody, and streptavidin-HRP (Vector Laboratories). Sections were visualized with DAB substrate (Vector Laboratories). Images were obtained using an Olympus CX31 microscope. Primary antibodies used in this study are as follows: 4-HNE (ab46545, Abcam), Ki67 (ab1667, Abcam), and Cleaved caspase-3 (9661, Cell Signaling Technology). For immunofluorescence staining, 786-O cells were cultured on glass-bottom dishes (Thermo Fisher), then treated with Salinomycin or DMSO. The cells were fixed and labeled with anti-LC3 and anti-PDIA4 primary antibodies and then exposed to Alexa 488 or 594-labeled secondary antibodies (Invitrogen). Images were visualized using an Olympus FV1000 confocal microscope.

### Assay of ferrous ion

Intracellular ferrous ion was determined using Iron Assay Kit (I291, Dojindo) following the manufacturer’s instructions. The values were measured using a microplate reader at the absorption wavelength of 593 nm. The iron concentration in tumor tissues was assessed using the Prussian Blue Iron Stain Kit (G1428, Solarbio Life Sciences) according to the manufacturer’s instructions. Images were obtained using an Olympus CX31 microscope.

### Xenograft mouse study

Six- to eight-week-old male BALB/c nude mice (Vital River, Beijing, China) were used for 786-O xenograft experiments. Briefly, 2 × 10^6^ 786-O cells in 200 μl PBS were implanted in the mice subcutaneously. Tumor growth was monitored regularly via external caliper measurements. When tumors reached the intended size, mice were divided randomly into two groups (*n* = 8): Vehicle group and Salinomycin group (5 mg/kg). Sal was dissolved in a solution of 10% DMSO and 90% corn oil. The usage of Sal was given as 5 mg/kg body weight per day intraperitoneally, and in total, nine times injection was administered. The maximum width (*a*) and length (*b*) of the tumor were measured every day, and the volume (*V*) was calculated using the formula: *V* = π × *a* × *b*^2^/6. Tumor growth was monitored over time. All animal experiments were approved by the University of Nankai’s Institutional Animal Use and Care Committee (2021-SYDWLL-000441). No blinding was done in the animal studies.

### RNA sequencing and identification of differentially expressed genes

Total RNA was extracted from 786-O-sc and 786-O-shPDIA4 stable cell lines using TRIzol reagent. RNA sequencing was performed and analyzed on the DNBSEQ platform (BGI, China). Gene expression was standardized to 1 million reads and normalized for gene length. The mRNAs with a fold change of >2 and <0.5 were considered upregulated and downregulated, respectively.

### Gene expression profiling, correlation, and survival analysis

PDIA4, ATF4, SLC7A11, and PERK expression were compared in Clear carcinoma (KIRC) and matched non-tumor kidney tissue by using the data from The Cancer Genome Atlas (TCGA, http://cancergenome.nih.gov) and Oncomine (http://www.oncomine.org) database. Pearson’s or Spearman’s correlation (two-sided) analysis was used to determine the expression correlation between PDIA4 and PERK or PDIA4 and PERK. The analysis of the correlation between the PDIA4 expression and the prognosis of KIRC patients was performed using the online Kaplan–Meier plotter tool (http://www.kmplot.com/).

### Patient samples

Human ccRCC specimens were obtained from patients of the Second Hospital of Tianjin Medical University who received nephrectomy treatment without radio- or chemotherapy (*n* = 11). Informed consent was obtained from human participants in this study. The tumor and adjacent normal tissues were dissected into two parts: one part of the tissues was fixed in 4% paraformaldehyde for histological studies; the other part was directly stored at −80 °C for RNA and protein extraction use. The case information of patients is listed in Supplementary Table 3. All studies involving human kidney sections were performed in accordance with the Chinese Ministry of Health national guidelines for biomedical research and approved by the Human Research Ethics Committee of Nankai University (NKUIRB2021078).

### Statistical analyses

All data are expressed as mean ± S.E.M. for experiments and numbers of mice used as indicated. The two-tailed Student’s *t*-test was used to evaluate statistical differences between the two groups. For multiple group comparisons, ANOVA followed by Bonferroni’s post-hoc correction was used. *P* values ≤ 0.05 was considered statistically significant. Statistical analysis of bar graphs and scatter plots in this manuscript was performed using Prism 7.0 software (GraphPad Software, San Diego, CA).

## Supplementary information

supplemental material

AJ-checklist

WB-RAW IMAGES

## Data Availability

The authors declare that all data supporting the findings of this study are available within the article or from the corresponding author on reasonable request.
