# Supplementary material for: PDIA4 confers resistance to ferroptosis via induction of ATF4/SLC7A11 in renal cell carcinoma
Source: Cell Death Dis. 2023 Mar 11;14(3):193. doi: 10.1038/s41419-023-05719-x (PMC10008556; doi:10.1038/s41419-023-05719-x)
Supplement: Supplementary file 1 — supplemental material [file 41419_2023_5719_MOESM1_ESM.docx]

PDIA4 Confers Resistance to Ferroptosis via Induction of ATF4/SLC7A11 in Renal Cell Carcinoma

**Lichun Kang**^1,2#^, **Dekun Wang**^1#^, **Tianyu Shen**^1^, **Bo Dai**^1^**, Huan Shen**^3^**, Xuan Liu**^1^**, Junbo Gong**^3^, **Gang Li**^4^, **Yuanjing Hu**^5^, **Peng Wang**^6^, **Xue Mi**^1^, **Yuying Zhang**^1^, **and Xiaoyue Tan^1^** *

**Supplementary Materials**

**Supplementary methods**

**2-D Colony Formation Assay**

Briefly, total 500 cells were seeded in 6-well plate. After 14 days, the cells were fixed in methanol and stained with 0.5% crystal violet in 25% methanol for 10 minutes. The cell plates were dried and then washed in 1% SDS. Cell density was quantified by measuring absorbance of the wash solution at 560 nm using a microplate reader.

**Supple****mentary figures**

**Supplementary fig 1.** Sal induces ferroptosis in breast cancer cell lines.

**Supplementary fig 2.** Expression of PDIA4 in the RCC cell lines and the samples form RCCs patients.

**Supplementary fig 3.** Silencing down PDIA4 inhibits cell growth of RCC cells.

**Supplementary fig 4**. Sal suppresses PDIA4 and thus ATF4/SLC7A11 in ACHN cells.

**Supplementary fig 5**. Expression of IRE1α and ATF6 in human RCC samples and cell lines.

**Supplementary tables**

**Supplementary table 1.** Primer sequences for shRNA construct

**Supplementary table 2**. Primer sequences for RT-PCR

**Supplementary table 3**. Personal information for human samples


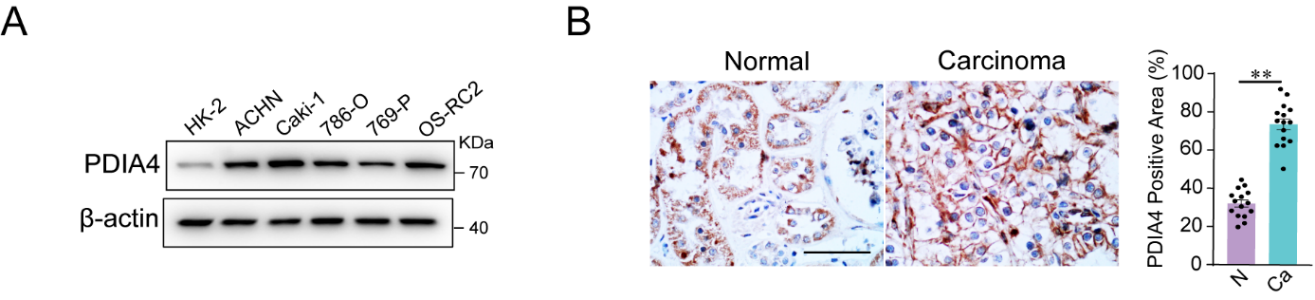
**
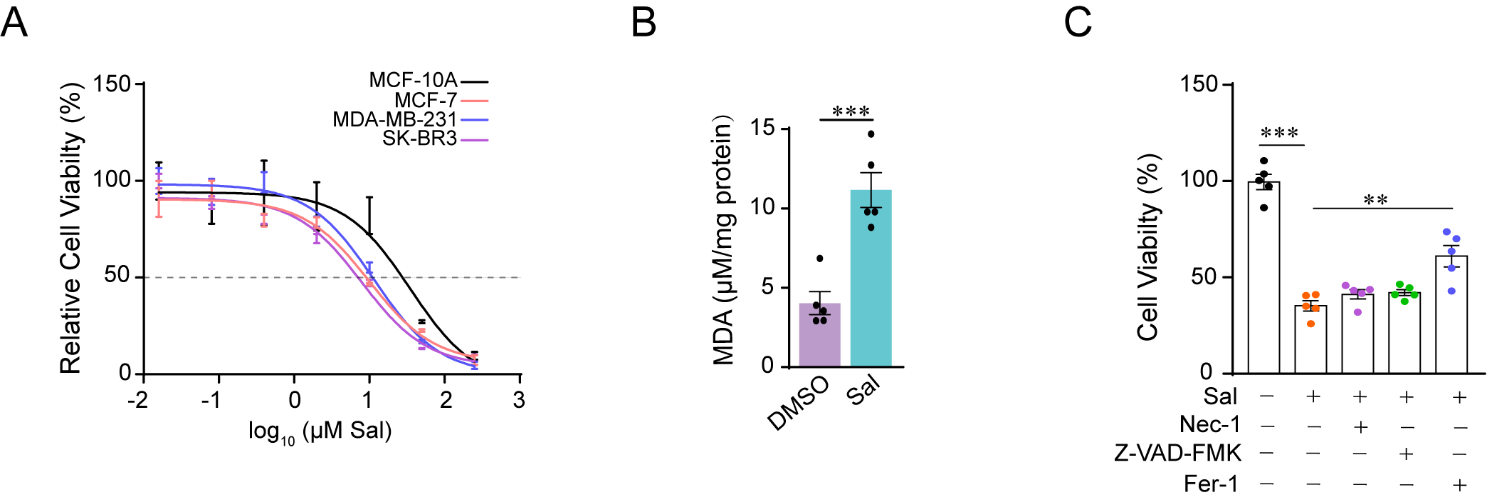
****Supplementary figure 1. Sal induces ferroptosis in breast cancer cell lines.** Human breast epithelial cell line MCF-10A, breast cancer cell line MCF-7, MDA-MB-231 and SK-BR3 were treated with different concentration of Sal as indicated for 48 hours. **(A)** Dose-response viability curves of cells treated with Sal. **(B)** MDA assay of the cell lysate from different groups. **(C)** Cell viability assay in MDA-MB-231 cells treated with Sal (2 µM) for 48 h, in the presence or absence of the indicated inhibitors. Data presents as mean values and S.E.M of three biological replicates. *P* values were calculated using Student’s *t*-test in A & B, and One-way ANOVA in C. **, *P*<0.01; ***, *P*< 0.001.


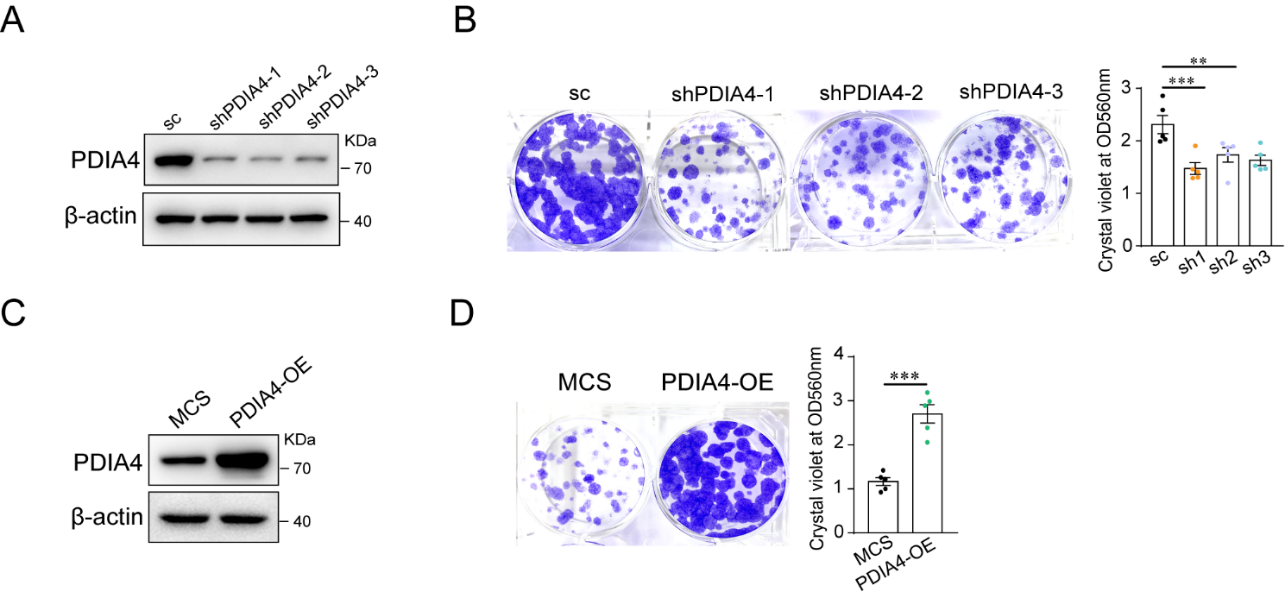
**Supplementary figure 2. Expression of PDIA4 in the RCC cell lines and the samples form RCCs patients.** RCC Specimens from patients were underwent immunohistological staining using the antibody against PDIA4 (n =11). The left panel is the representative images of tumor adjacent normal tissue and carcinoma tissue. Statistical data are shown in the right panel. Data presents as mean values and S.E.M of three biological replicates. Student’s *t*-test. **, *P*<0.01.

**Supplementary figure 3. Silencing down PDIA4 inhibits cell growth of RCC cells.** Stable PDIA4- or control MCS- overexpressing 786-O cells were established. **(A)** Western blot analysis of PDIA4 expression in sc, shPDIA4-1, shPDIA4-2 and shPDIA4-3 786-O cells. **(B)** Representative images (left) and quantification (right) of 2D colonies. **(C)** Western blot analysis of PDIA4 expression in MCS, PDIA4-Over Expressing (OE) 786-O cells. **(D)** Representative images (left) and quantification (right) of 2D colonies. Data presents as mean values and S.E.M of three biological replicates. Student’s *t*-test. **, *P*<0.01. ***, *P*< 0.001


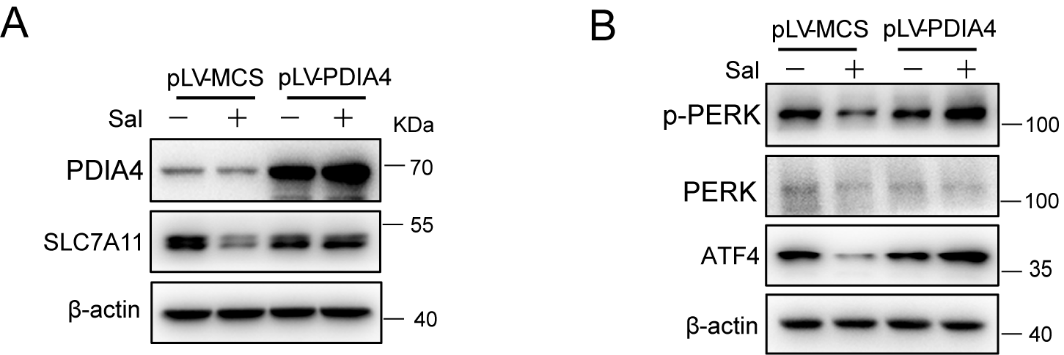


**Supplementary figure 4. Sal suppresses PDIA4 and thus ATF4/SLC7A11 in ACHN cells.** Stable PDIA4- or control MCS- overexpressing ACHN cells were treated with Sal (2 µM) for 48 hours. **(A)** Western blot assay using antibody against PDIA4 and SLC7A11. **(B)** Western blot assay using antibody against p-PERK, PERK and ATF4.


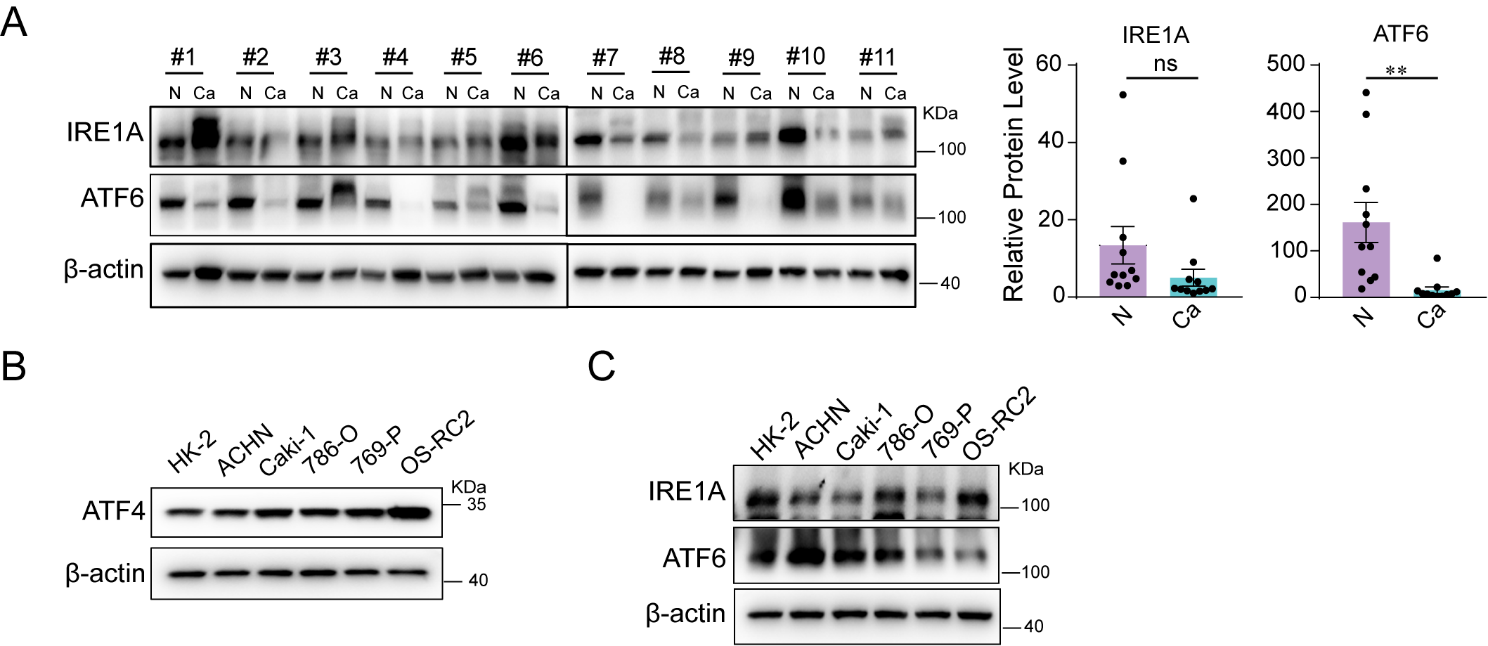


**Supplementary figure 5. Expression of IRE1a and ATF6 in human RCC samples and cell lines.** Human renal carcinoma and corresponding paracancerous tissues were obtained from patients of renal carcinoma underwent nephrectomy. **(A)** Western blot assay of tissue homogenates using antibody against IRE1α and ATF6. The lower panel shows the statistical results. **(B)** Western blot assay of ATF4 and **(C)** IRE1α and ATF6 in human proximal tubular cell line HK-2 and RCC cell lines ACHN, Caki-1, 786-O, 769-p and OSRC2. Statistical data are shown in the right panel. Data presents as mean values and S.E.M of three biological replicates. Student’s *t*-test. **, *P*<0.01.

**Supplementary table 1. Primer sequences for shRNA**

| shRNAs | Primer Sequence |
| --- | --- |
| shPDIA4-1 | F: CCGGCCTGAGAGAAGATTACAAATTCTCGAGAATTTGTAATCTTCTCTCAGGTTTTTG |
|  | R: AATTCAAAAACCTGAGAGAAGATTACAAATTCTCGAGAATTTGTAATCTTCTCTCAGG |
| shPDIA4-2 | F: CCGGGCTTGTGTTGACCAAAGAGAACTCGAGTTCTCTTTGGTCAACACAAGCTTTTTG |
|  | R: AATTCAAAAAGCTTGTGTTGACCAAAGAGAACTCGAGTTCTCTTTGGTCAACACAAGC |
| shPDIA4-3 | F: CCGGCTTGGTCCTAAATGATGCAAACTCGAGTTTGCATCATTTAGGACCAAGTTTTTG |
|  | R: AATTCAAAAACTTGGTCCTAAATGATGCAAACTCGAGTTTGCATCATTTAGGACCAAG |
| shATG5-1 | F: CCGGCCTGAACAGAATCATCCTTAACTCGAGTTAAGGATGATTCTGTTCAGGTTTTTG |
|  | R: AATTCAAAAACCTGAACAGAATCATCCTTAACTCGAGTTAAGGATGATTCTGTTCAGG |
| shATG5-2 | F: CCGGCCTTTCATTCAGAAGCTGTTTCTCGAGAAACAGCTTCTGAATGAAAGGTTTTTG |
|  | R: AATTCAAAAACCTTTCATTCAGAAGCTGTTTCTCGAGAAACAGCTTCTGAATGAAAGG |

**Supplementary table 2. Primer sequences for RT-PCR**

| Genes | Primer-Forward | Primer-Reverse |
| --- | --- | --- |
| *PDIA4* | AAGCGTTCTCCTCCAATT | GGACTGCTCGATCATGTAA |
| *SLC7A11* | TCCTGCTTTGGCTCCATGAACG | AGAGGAGTGTGCTTGCGGACAT |
| *β-actin* | CAGAAGGAGATTACTGCTCTGGCT | TACTCCTGCTTGCTGATCCACATC |

**Supplementary table 3. Personal information for human samples**

| NO. | Ages (Years Old) | Sex | Pathologic Diagnosis | pTNM |
| --- | --- | --- | --- | --- |
| 1 | 36 | Male | ccRCC | pT1aNxMx |
| 2 | 66 | Female | ccRCC | pT2NxMx |
| 3 | 56 | Male | ccRCC | pT3aNxMx |
| 4 | 31 | Female | ccRCC | pT1aNxMx |
| 5 | 61 | Male | ccRCC | pT2bNxMx |
| 6 | 32 | Female | ccRCC | pT1aNxMx |
| 7 | 60 | Male | ccRCC | pT3aNxMx |
| 8 | 41 | Female | ccRCC | pT1aNxMx |
| 9 | 38 | Male | ccRCC | pT1aNxMx |
| 10 | 68 | Female | ccRCC | pT2NxMx |
| 11 | 59 | Female | ccRCC | pT1aNxMx |
