# Supplementary material for: PDIA4 confers resistance to ferroptosis via induction of ATF4/SLC7A11 in renal cell carcinoma
Source: Cell Death Dis. 2023 Mar 11;14(3):193. doi: 10.1038/s41419-023-05719-x (PMC10008556; doi:10.1038/s41419-023-05719-x)

Figure 1E

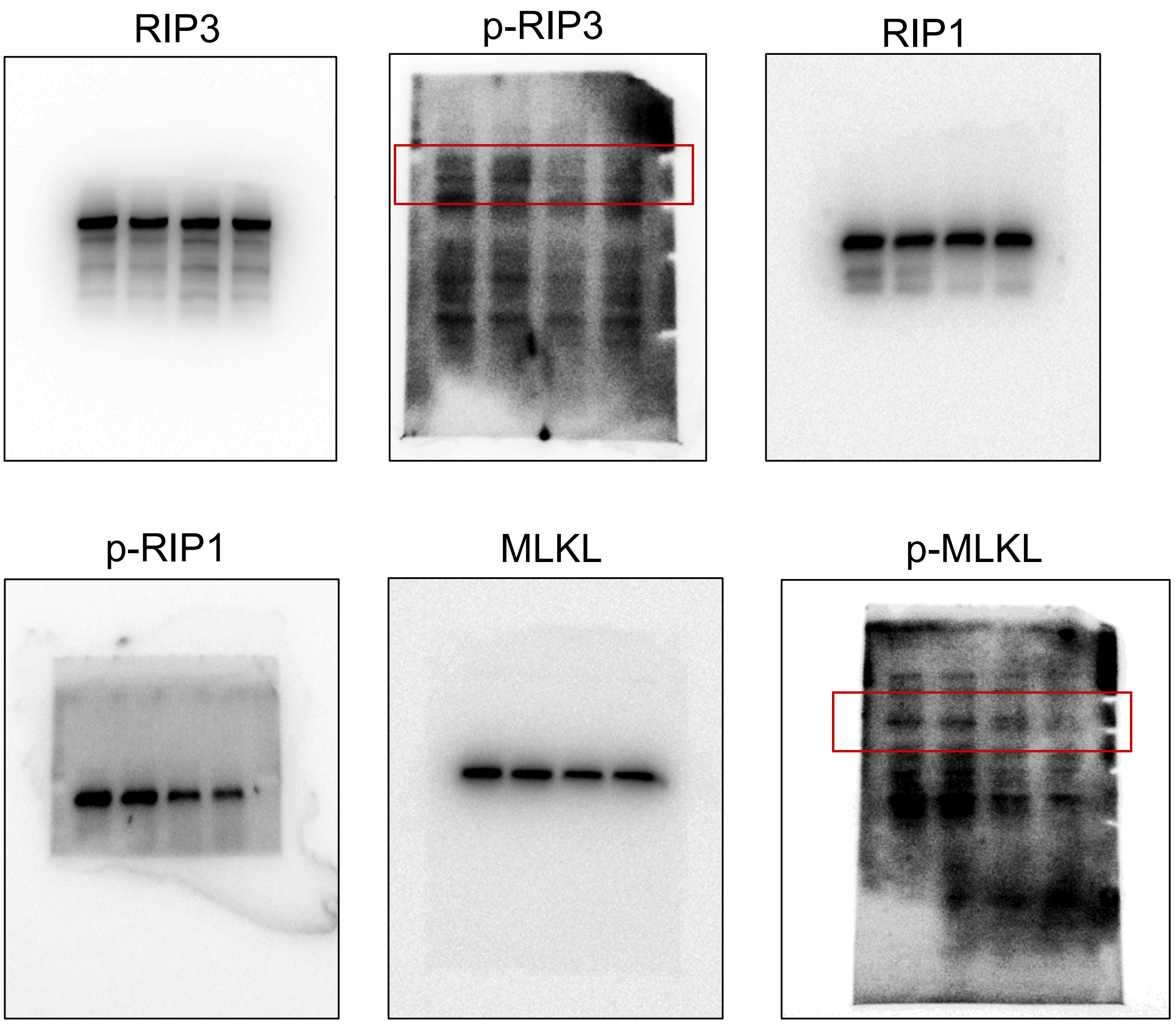

Figure 1E

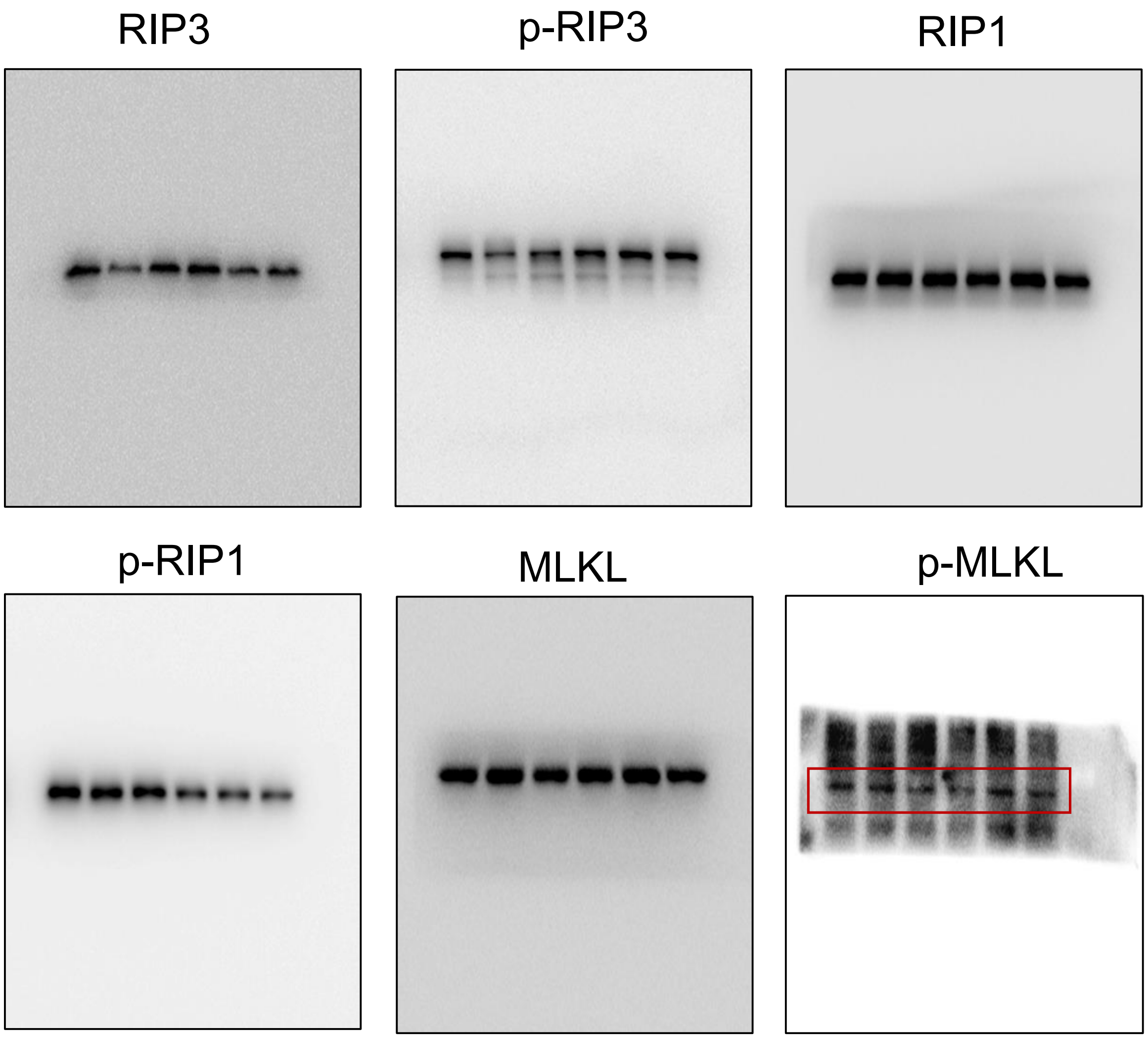

Figure 1F

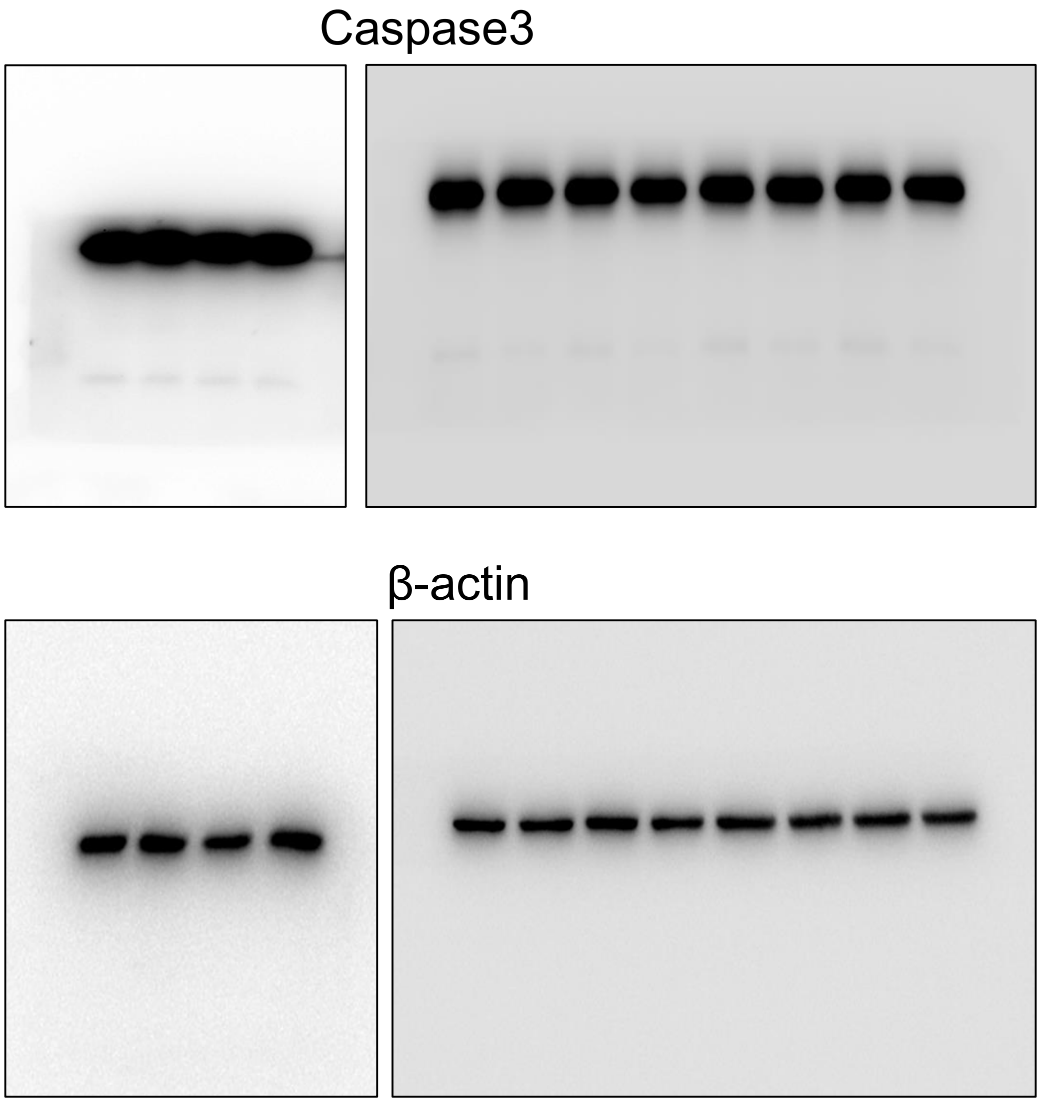

Figure 2C

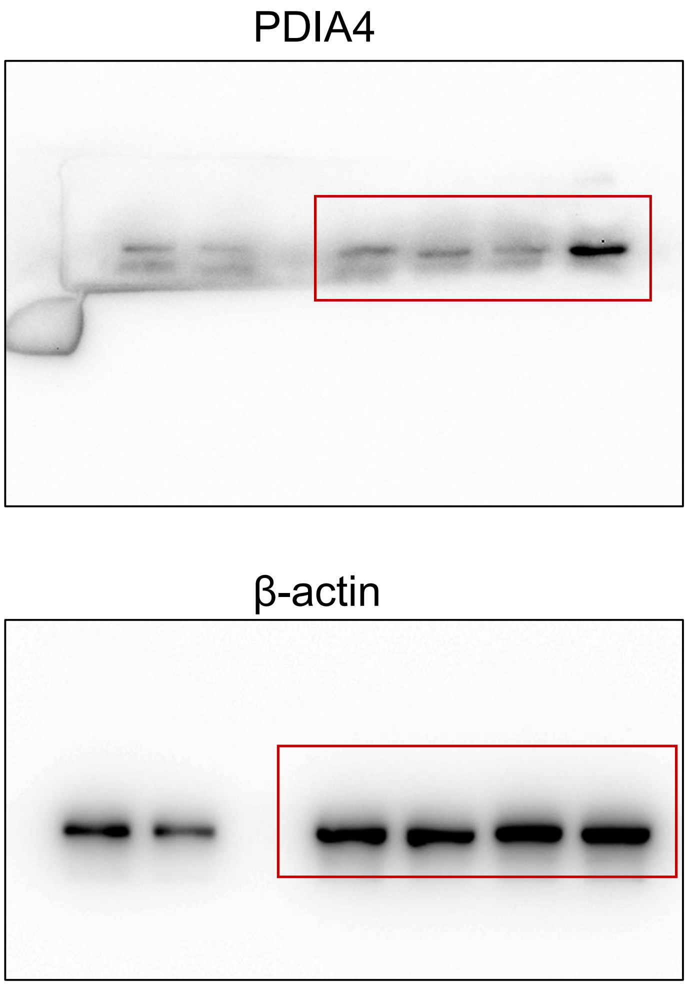

Figure 2D

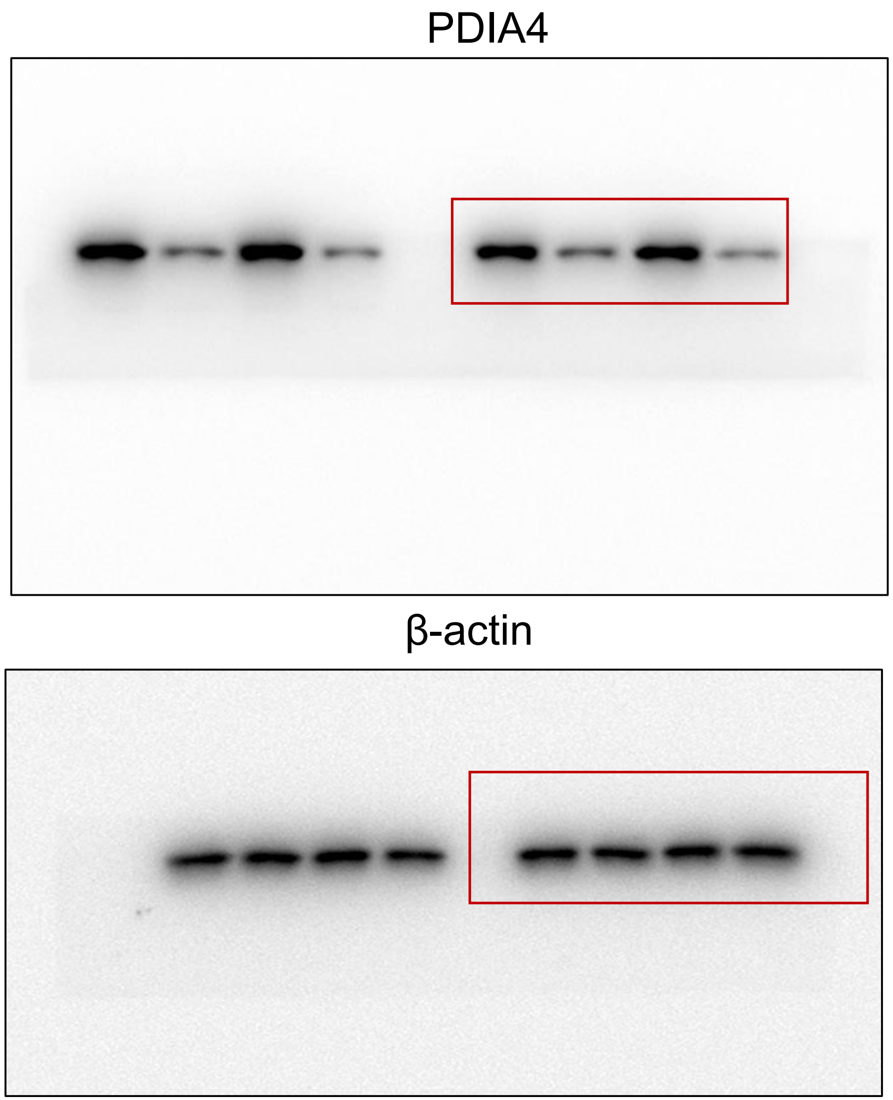

Figure 2E

PDIA4

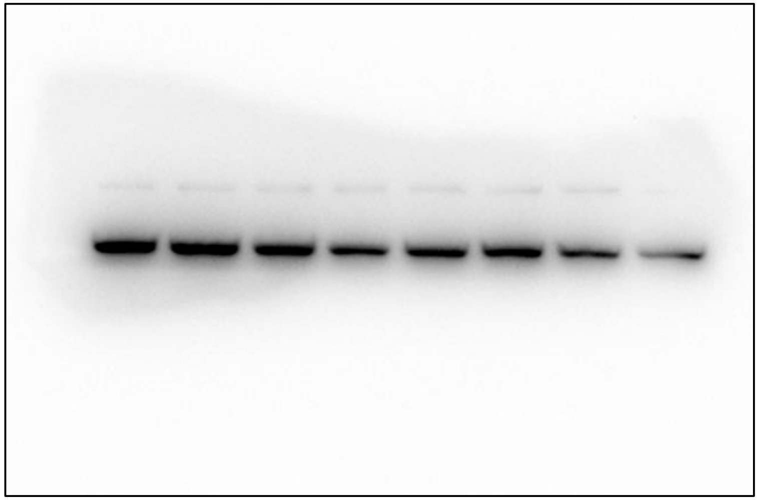

β-actin

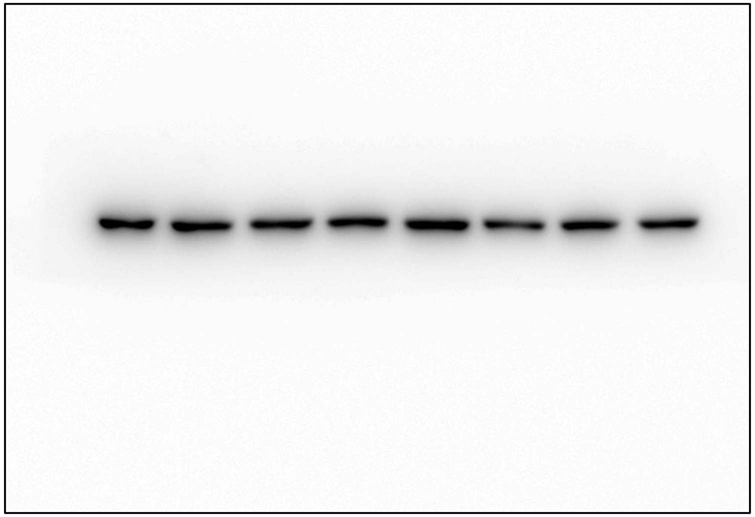

Figure 2F

PDIA4

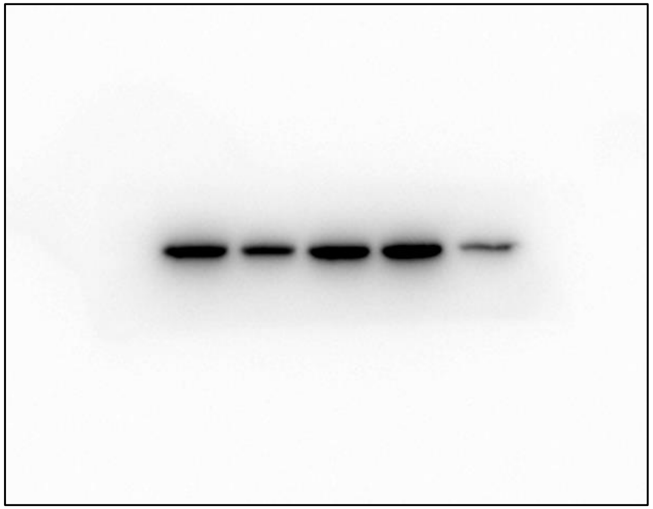

β-actin

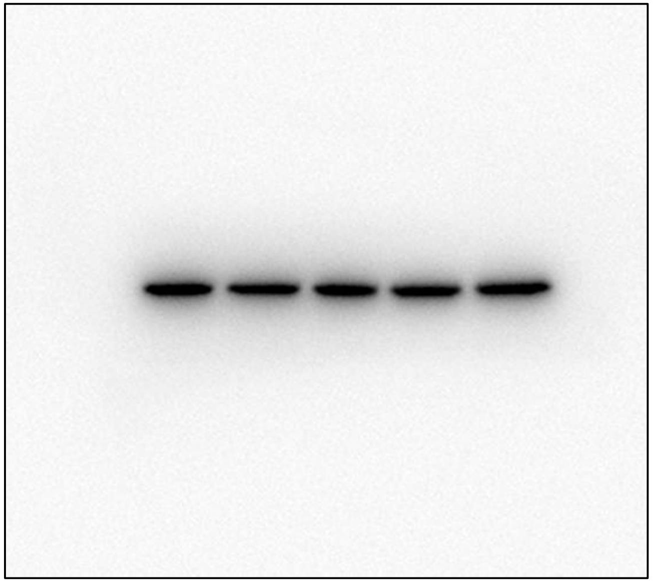

Figure 2H

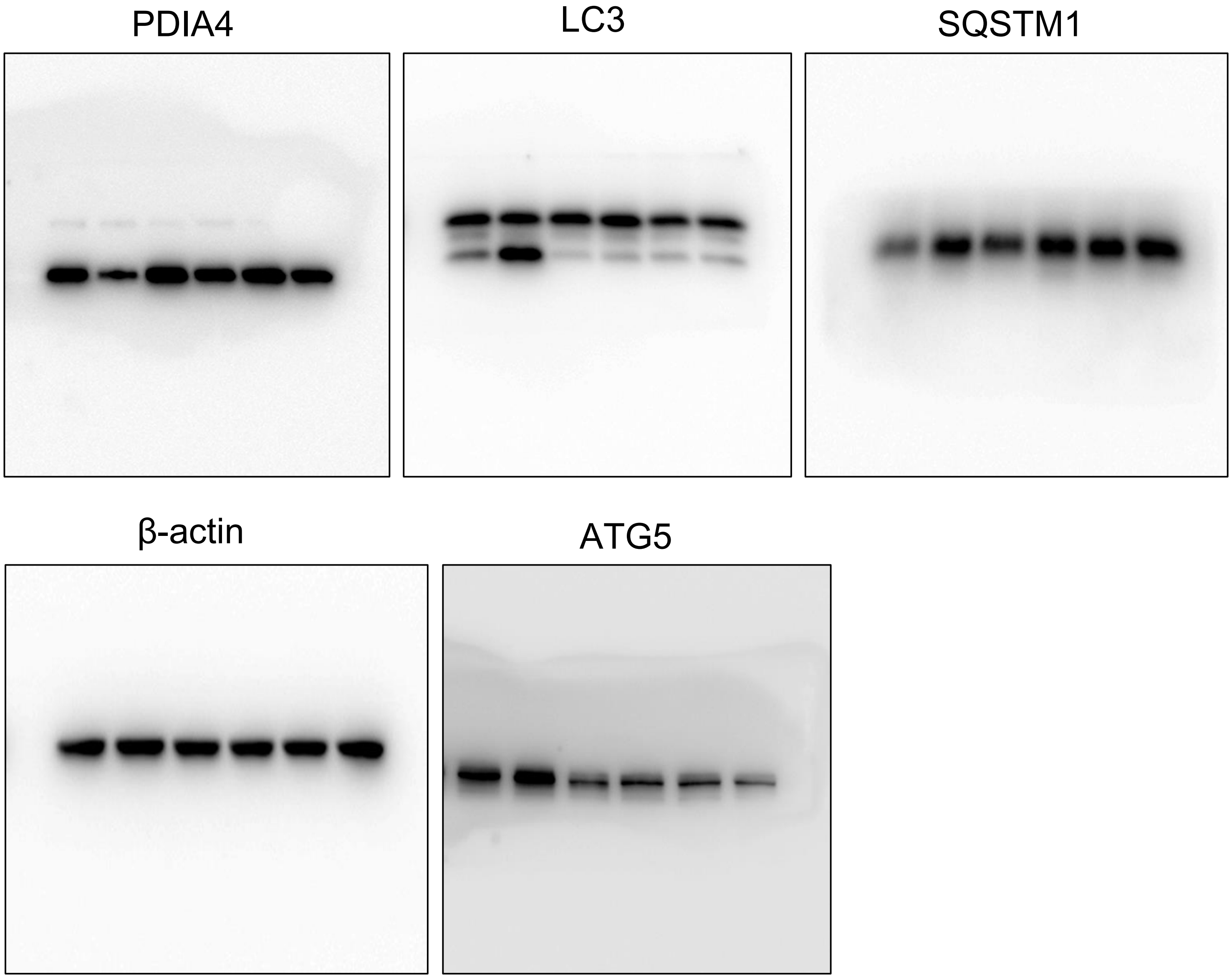

Figure 2I

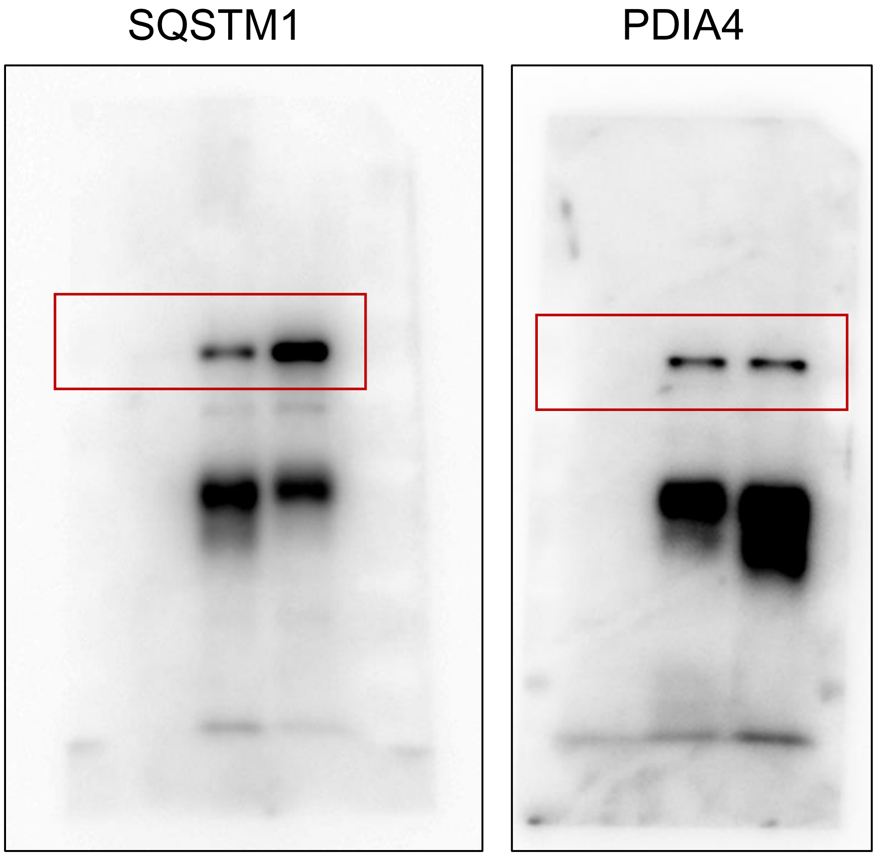

Figure 3A

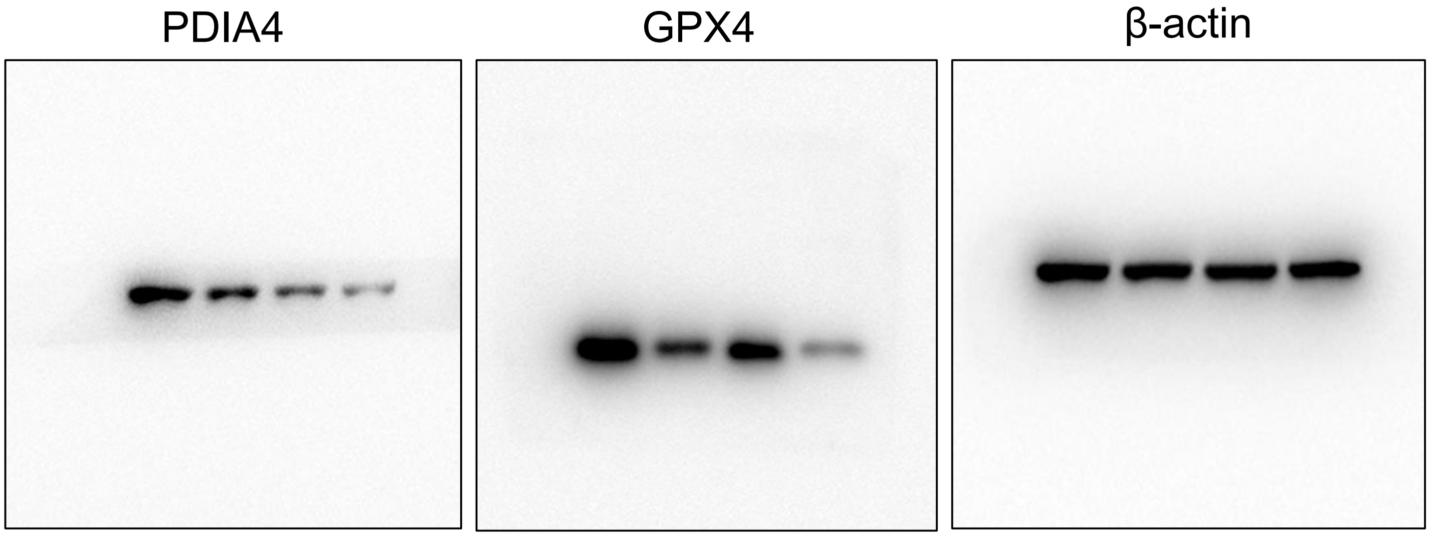

Figure 3G

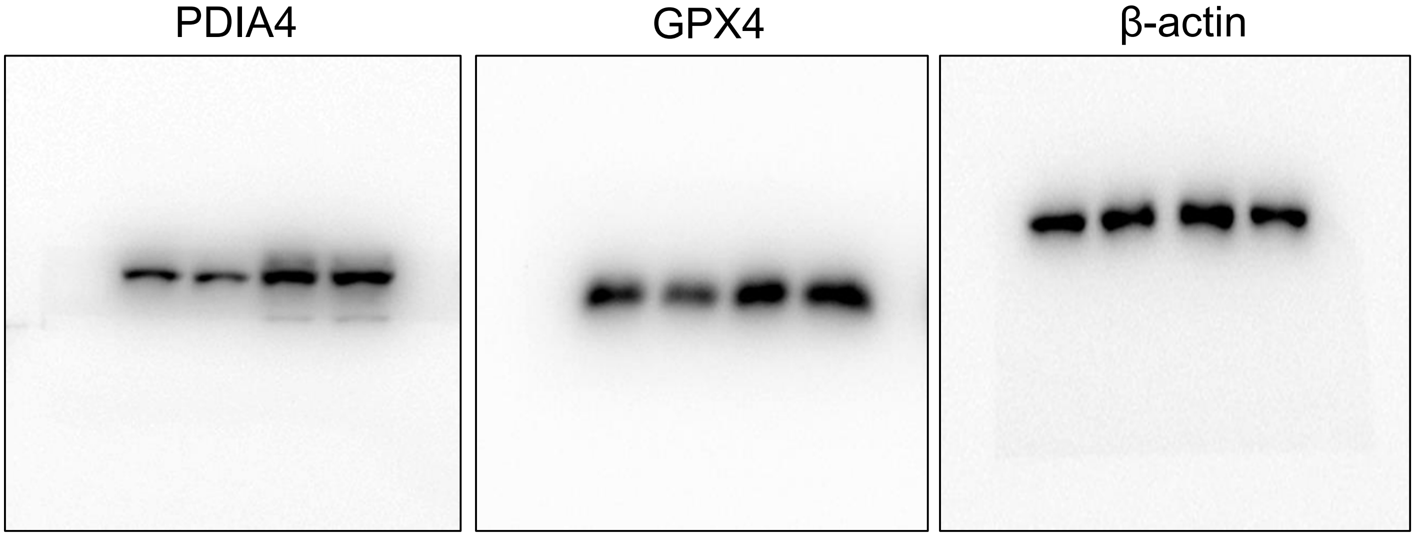

Figure 4C

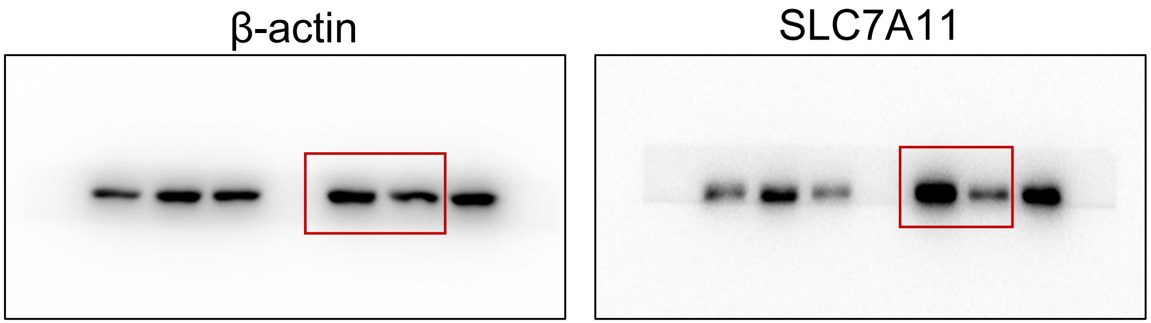

Figure 4D

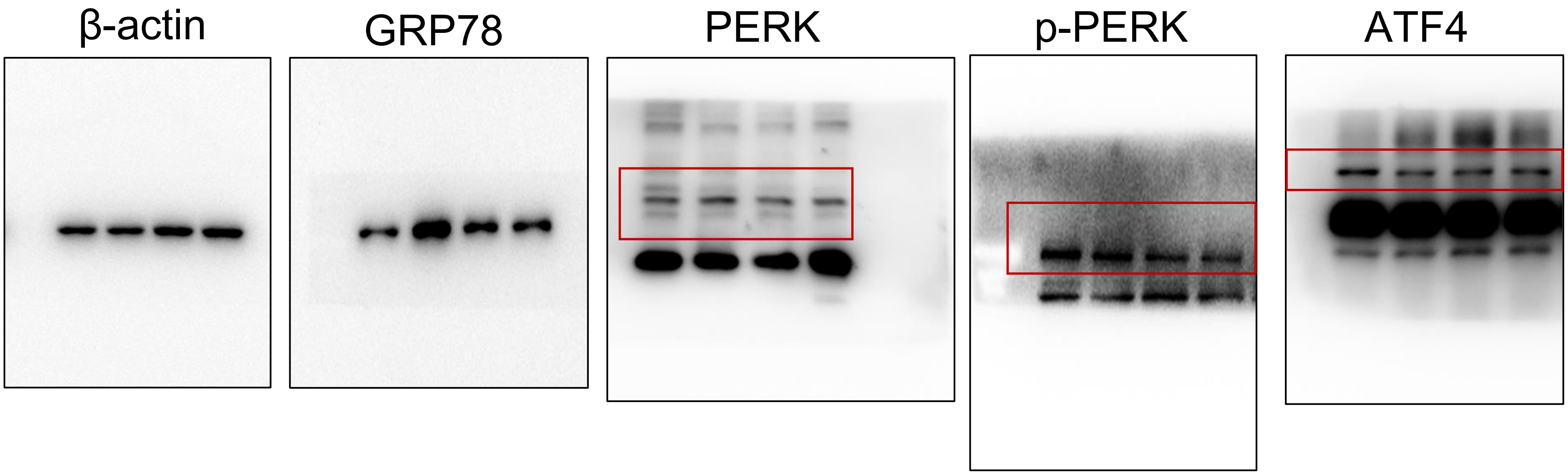

Figure 4E

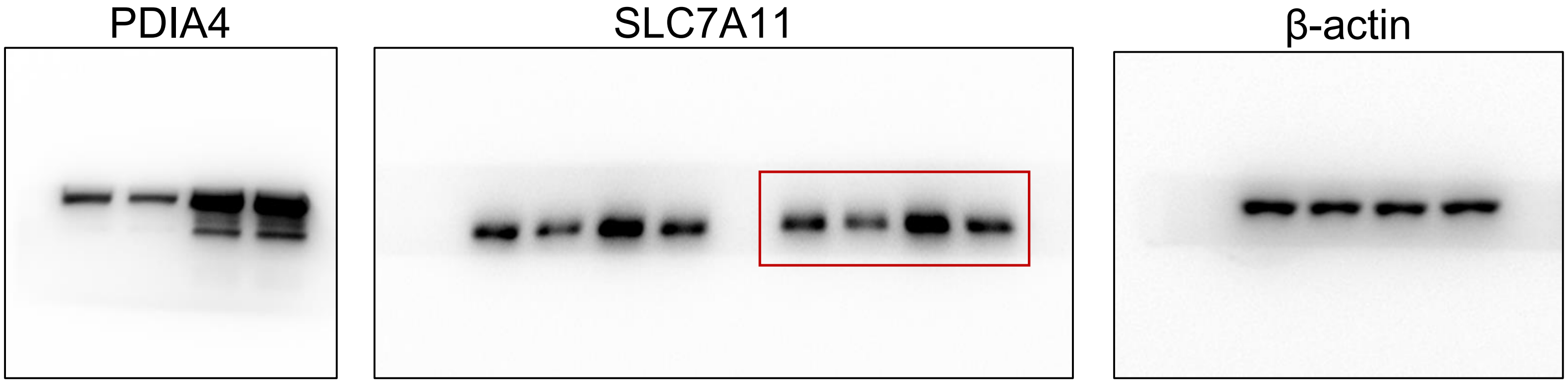

Figure 4F

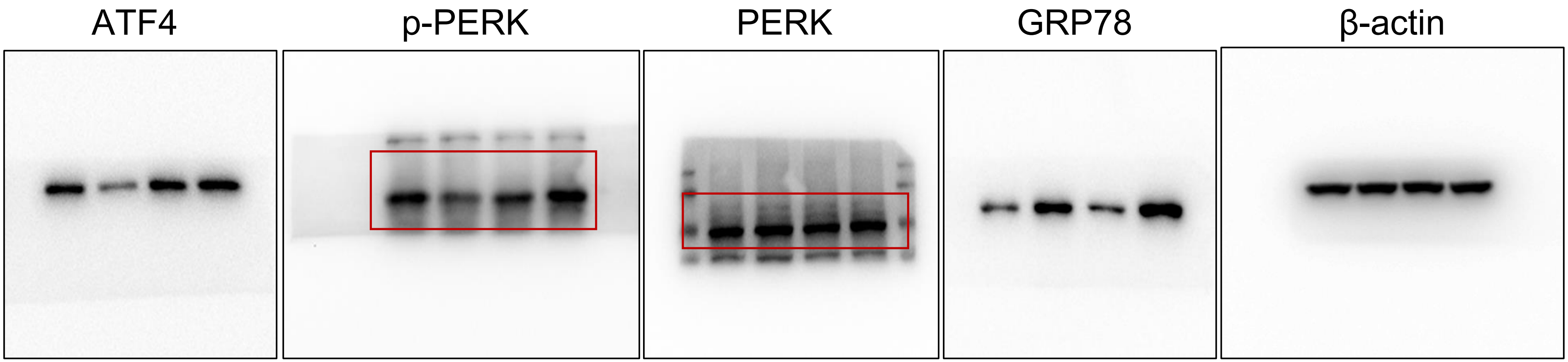

Figure 4I

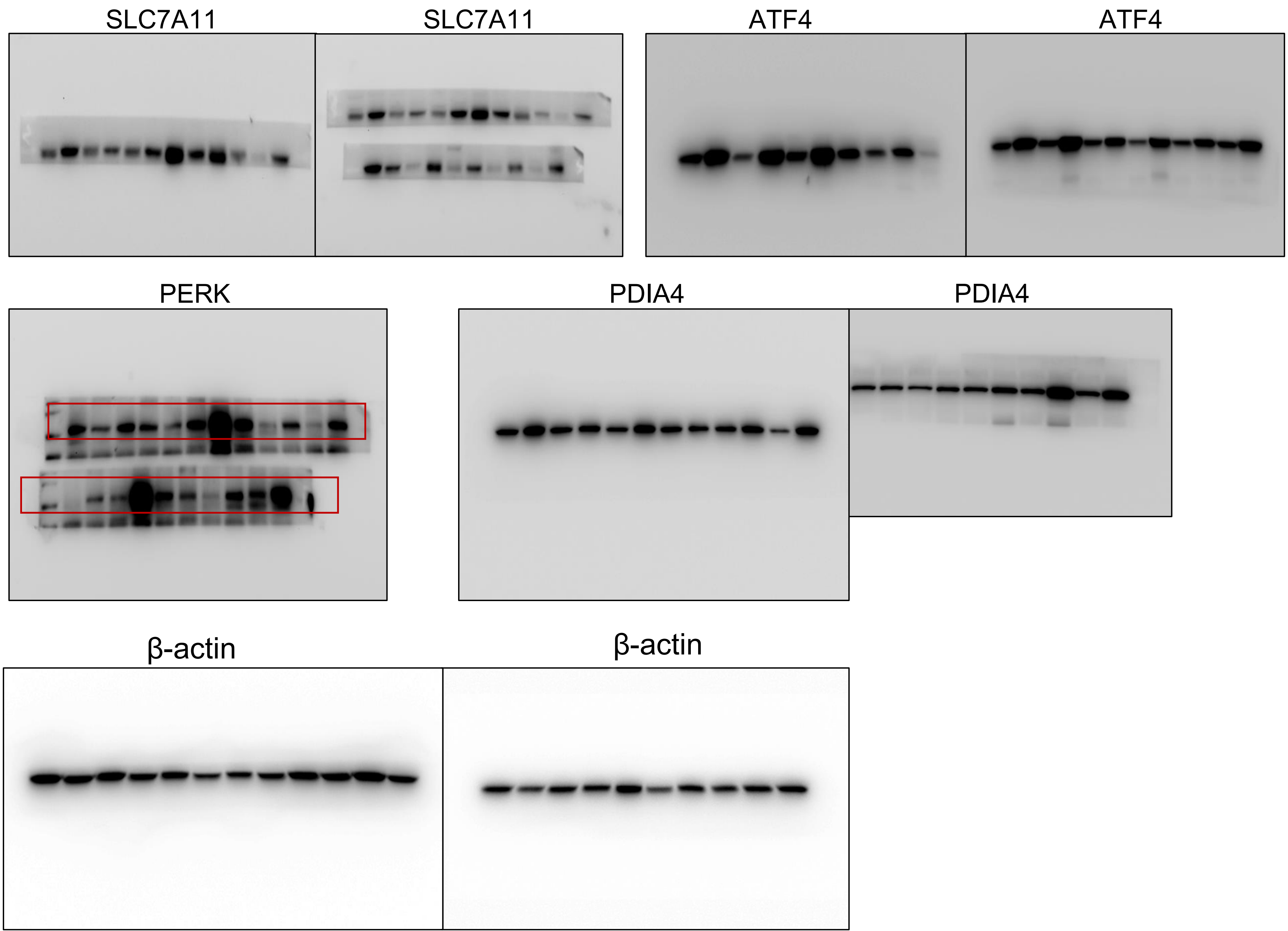

Figure 5H

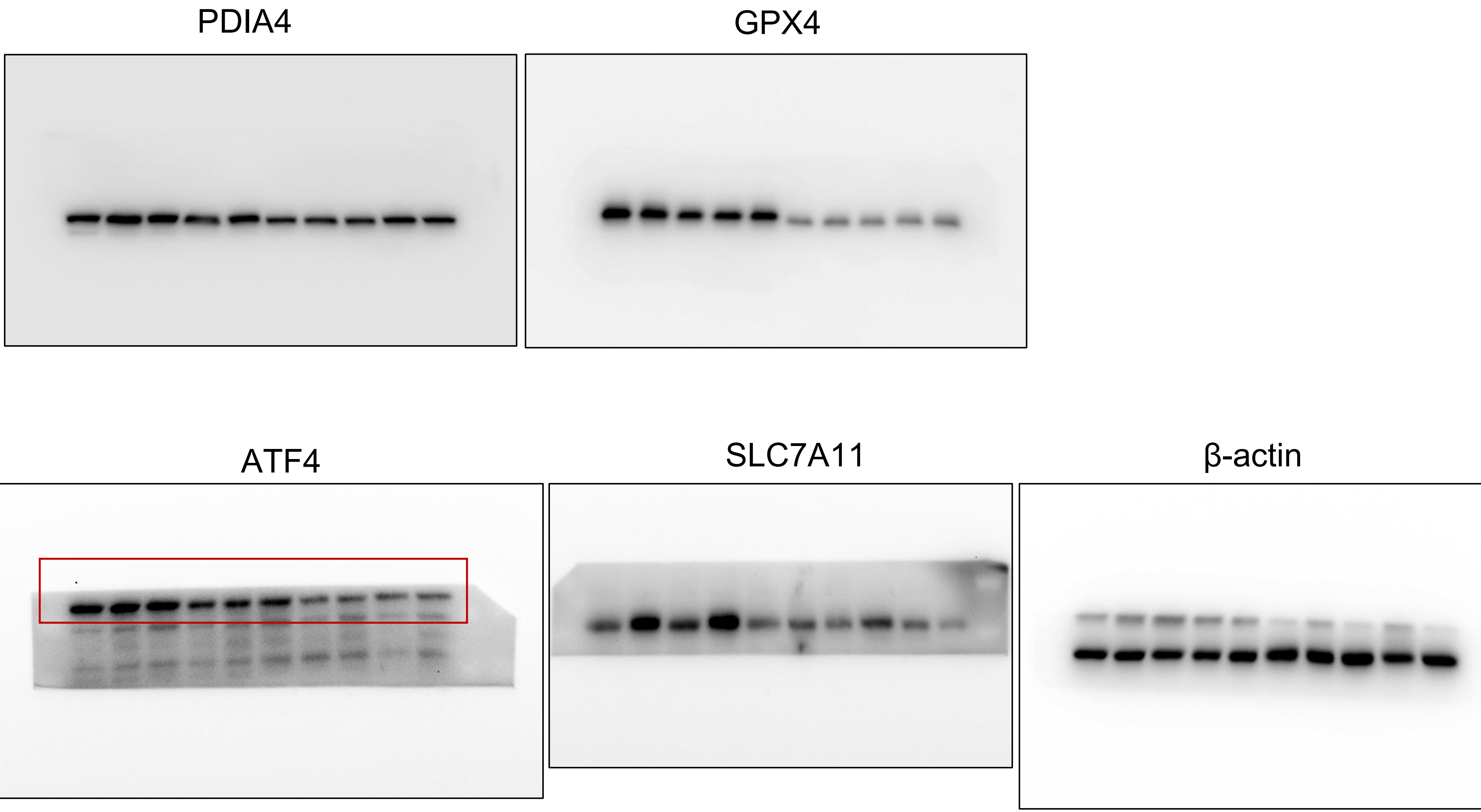

Figure S2

PDIA4

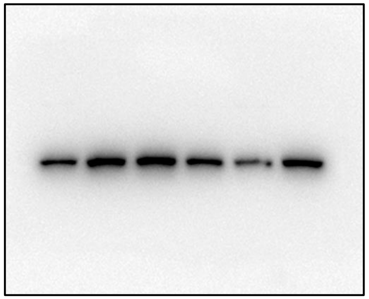

β-actin

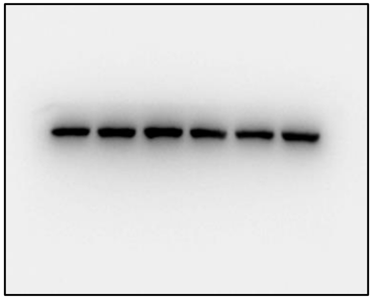

Figure S3A

PDIA4

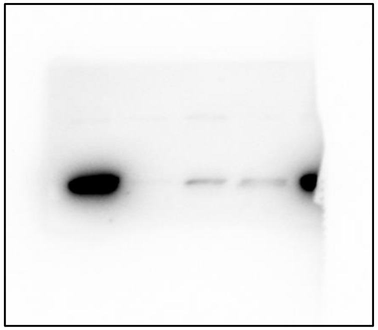

β-actin

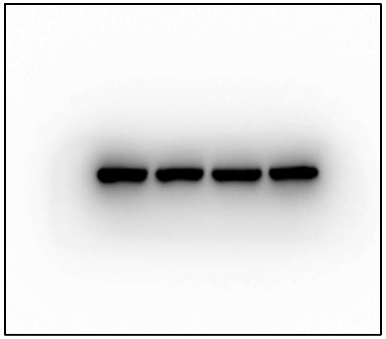

Figure S3C

PDIA4

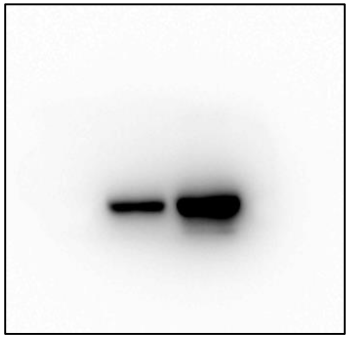

β-actin

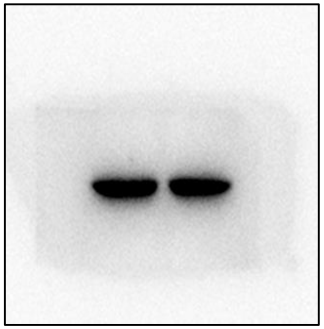

Figure S4A

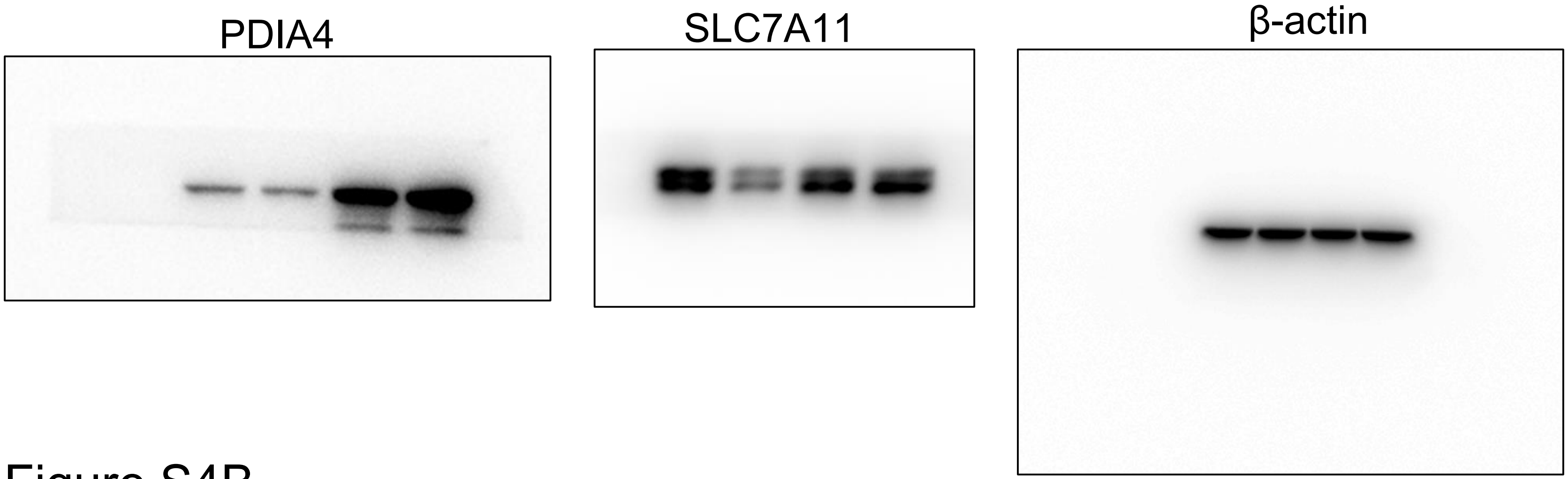

Figure S4B

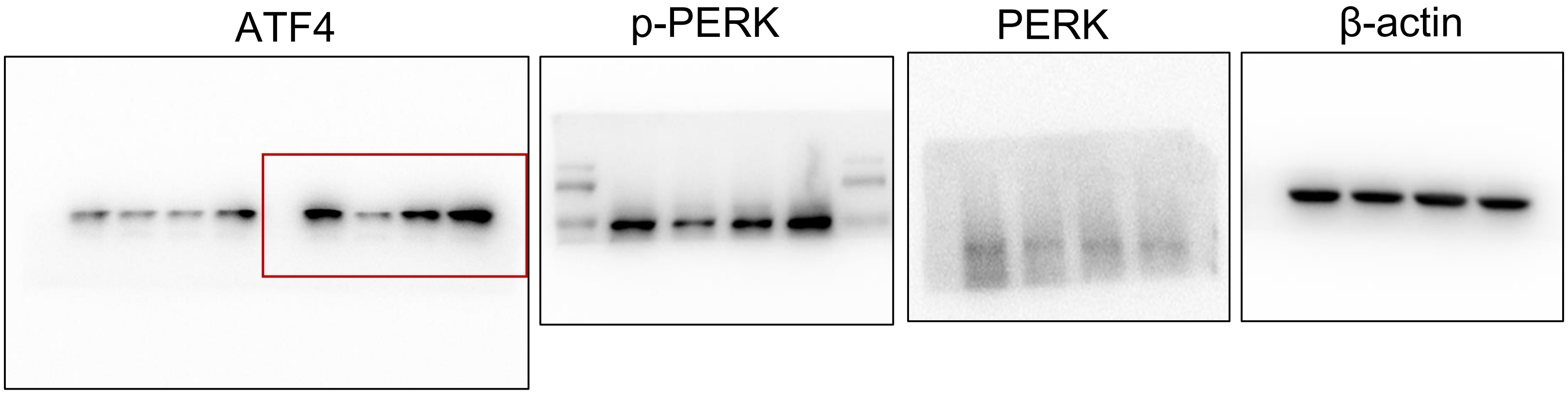

Figure S5A

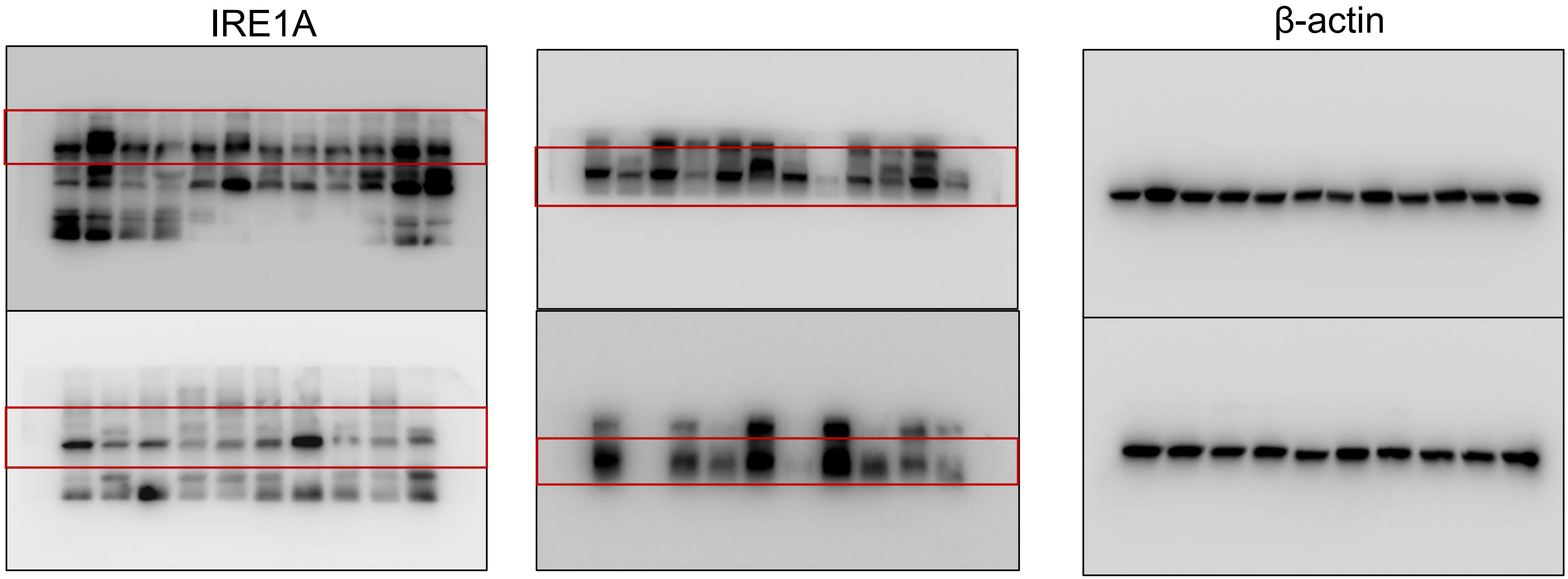

Figure S5B&C

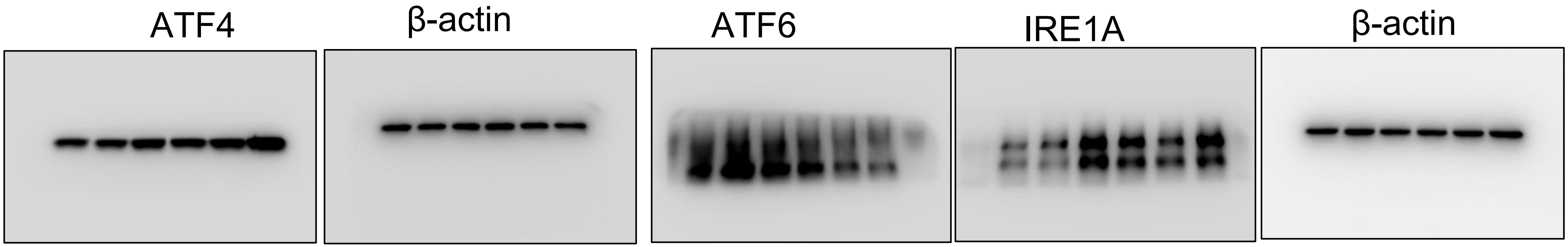

Supplement: Supplementary file 3 — WB-RAW IMAGES [file 41419_2023_5719_MOESM3_ESM.pdf]
